# Identification of Metabolites and Antioxidant Constituents from *Pyrus ussuriensis*

**DOI:** 10.3390/ph19010192

**Published:** 2026-01-22

**Authors:** Ducdat Le, Thientam Dinh, Soojung Yu, Yun-Jin Lim, Hae-In Lee, Jin Woo Park, Deuk-Sil Oh, Mina Lee

**Affiliations:** 1 College of Pharmacy and Research Institute of Life and Pharmaceutical Sciences, Sunchon National University, 255 Jungangno, Suncheon 57922, Jeonnam, Republic of Korea; ddle@scnu.ac.kr (D.L.); 1243010@s.scnu.ac.kr (T.D.); 2 Department of Natural Cosmetics Science, Graduate School, Sunchon National University, 255 Jungangno, Suncheon 57922, Jeonnam, Republic of Korea; sjyu@scnu.ac.kr; 3 Jeollanamdo Forest Research Institute, Naju 58213, Jeonnam, Republic of Korea; dladbswls@korea.kr (Y.-J.L.); ohye@korea.kr (D.-S.O.); 4 Department of Food and Nutrition, Sunchon National University, 255 Jungangno, Suncheon 57922, Jeonnam, Republic of Korea; leehi3657@scnu.ac.kr; 5 Department of Biomedicine, Health & Life Convergence Sciences, BK21 Four, Biomedical and Healthcare Research Institute, Mokpo National University, Mokpo 58554, Jeonnam, Republic of Korea; jwpark@mokpo.ac.kr; 6 College of Pharmacy and Natural Medicine Research Institute, Mokpo National University, Mokpo 58554, Jeonnam, Republic of Korea

**Keywords:** *Pyrus ussuriensis*, LC-MS, molecular network, metabolites, LC-DPPH, antioxidants, ADME

## Abstract

**Background/Objectives**: *Pyrus ussuriensis* Maxim. has been cultivated in many regions worldwide. This plant is also regarded as a profitable fruit crop for the development of many food and functional products. There is limited research on the application of the LC-MS associated reaction method for screening active compounds. In this study, we developed an analytical technique employing an ultra-high-performance liquid chromatography-electrospray ionization-tandem mass spectrometry (UHPLC-ESI-MS/MS) system. **Methods**: The metabolite annotation procedure was used to interpret and validate data analysis via spectral matching against public databases. **Results**: As a result, metabolites from *P. ussuriensis* water and EtOH extracts were identified, and their quantities were further evaluated. The established method was employed to determine antioxidant capacity using a pre-incubation UHPLC-2,2-diphenyl-1-picrylhydrazyl (DPPH) assay, thereby identifying antioxidant ingredients. The antioxidative interference of active constituents was predicted by calculating the decrease in the peak areas of the chemical composition detected in chromatograms between treated and non-treated samples. Furthermore, drug-likeness was also assessed via pharmacokinetics (absorption, distribution, metabolism, and excretion: ADME) evaluation. **Conclusions**: The online UHPLC-MS-DPPH method would be a powerful tool for the rapid characterization of antioxidant ingredients in plant extracts. The current study highlights the value of *P. ussuriensis* for improved health benefits.

## 1. Introduction

*Pyrus ussuriensis* belongs to the genus *Pyrus* and is known as an economic fruit crop [1]. Thus, it has been planted in many regions worldwide. It is a valuable plant material for studying its chemical composition and antioxidant activity because of its great flavor, drought tolerance, and delicate, disease-resistant traits [2,3,4]. It has also been used for millennia in traditional Chinese folk medicine due to its antitussive, phlegm-dispelling, and diuretic properties. Additionally, *P. ussuriensis* showed an inflammatory effect in the lung by inhibiting myeloperoxidase activity and reducing the production of pro-inflammatory cytokines, thereby inactivating the mitogen-activated protein kinase (MAPK) and nuclear factor erythroid-2-related factor 2/heme oxygenase-1 (Nrf2/HO-1) signaling pathways. On the other hand, the extract from *P. ussuriensis* was found to decrease the ulcer index by preventing gastric mucosal lesions, erosion, and cellular degeneration [2]. Its extract also revealed antiproliferative effects against Bel-7402 cells. In particular, the isolated compound from this plant may induce apoptosis via the caspase pathway and cause cell cycle arrest at the G0/G1 phase by inhibiting the cyclin D1/CDK4 pathway [2]. Notably, *P. ussuriensis* has a high phenolic content, demonstrating antioxidant capacity in extracts, fractions, and active components. Previous studies have shown that extracts from *P. ussuriensis* peels and fruits exhibit antioxidative capacity [3,5]. However, there is no report on the development of an effective methodology for applying DPPH-based LC systems to determine antioxidants from this plant to date.

LC-MS is a highly efficient analytical technique that combines the separation capabilities of liquid chromatography with mass spectrometry detection. LC-MS is a powerful analytical instrument with optimal characteristics, such as high sensitivity, versatility, accurate capacity, and high chromatographic resolution. Furthermore, because it is simple to use and operate, LC-MS is well known for its utility and convenience [6]. With the above-mentioned benefits, it can also develop accurate methods by quantifying diversity in combination with many integrated devices, depending on the purpose of use, to reduce the expense and duration of experimental procedures across a variety of industrial applications in cosmetics, pharmaceuticals, food, drugs, and biotechnology.

In this study, we established an analytical method using the UHPLC-MS/MS system to determine the chemical constituents from water and EtOH extracts of *P. ussuriensis*. By combining modern mass spectrometry methods, we identified the chemicals present in both water and EtOH extracts of *P. ussuriensis* through MS/MS dereplication strategies together with simultaneous mass spectrometry analysis in conjunction with open web platforms. From there, the active compounds and other constituents of extracts have been identified with the difference in mass spectral signals less than 5 mDa. As a result, 150 secondary metabolites were identified by analyzing their ion precursors, mass fragmentation, and spectral matching with public mass spectrometry databases and the GNPS web platform. To discover the active compounds targeting antioxidants, we established an LC-MS method to determine the antioxidative capacity of *P. ussuriensis* by applying the LC-DPPH method. The established method was to evaluate antioxidant activity by identifying active components in *P. ussuriensis* extracts. Subsequently, an in silico study was conducted to assess the antioxidative potential of the major ingredients of both P. ussuriensis extracts using docking scores from binding complexes.

## 2. Results and Discussion

### 2.1. Molecular Network Guided Identification of Metabolites from Extracts of P. ussuriensis

**Figure 1:**
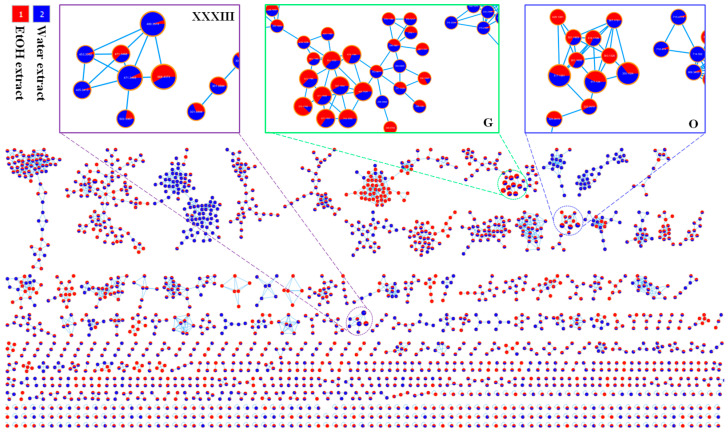
Feature-based molecular network of water (Blue) and EtOH (Red) extracts of *P. ussuriensis*. The G cluster showed the presence of flavonoid compounds. The O cluster displayed presence of quinic compounds. The XXXIII cluster demonstrated the fragments of glycosides.

**Figure 2:**
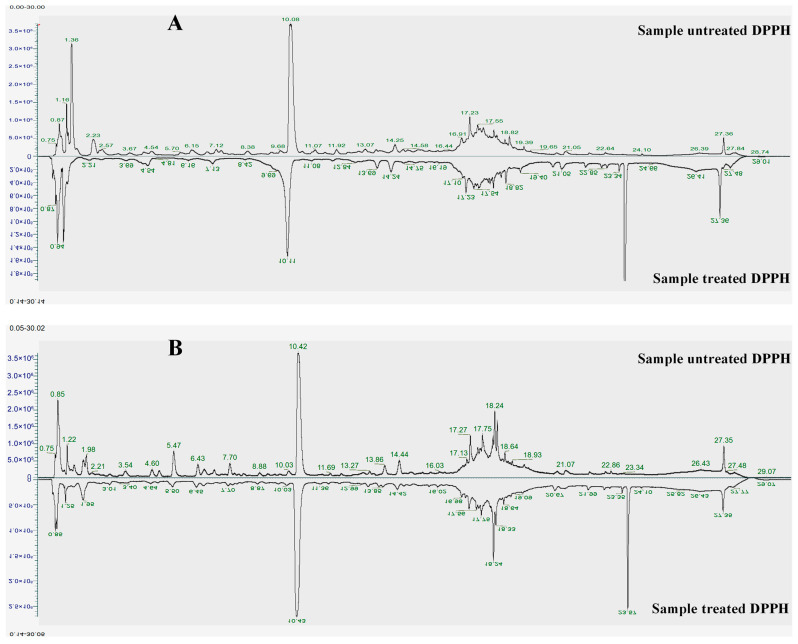
Chromatograms of the sample, untreated and treated with DPPH of water (**A**) and EtOH (**B**) extracts of *P. ussuriensis*. The active compounds were determined by integrating the peak areas of chromatograms obtained at the same retention time and mass spectra for the reaction mixture and untreated DPPH samples. Significant compounds were identified by reducing the original peak areas by more than 10% in untreated DPPH samples.

The feature-based molecular network built 305 clusters, which were classified by grouping compounds composed of molecules with similar fragmentation patterns (Figure 1). As shown in Figure 2, cluster G displayed nodes of flavonoid compounds. Briefly, their precursor ion peaks at *m*/*z* 743.2035 [M+H]^+^, 627.1557 [M+H]^+^, 611.1608 [M+H]^+^, 597.1452 [M+H]^+^, 551.1032 [M+H]^+^, and 465.1027 [M+H]^+^ were observed and displayed the same fragment at about *m*/*z* 303.10 [Quercetin+H]^+^, suggesting that they are quercetin derivatives. Analysis of mother patterns and fragmentation producing daughter ion peaks allowed us to identify those as quercetin-3-(2*R*-apiosylrutinoside), quercetin 3,4′-diglucosides, rutin, quercetin-3-*O*-(6-*O*-xylopyranosyl)-*β*-D-glucoside, quercetin-3-*O*-(6″-*O*-malonyl)glycoside, and quercetin-3-*O*-*β*-D-glucoside, respectively. Additionally, the precursor ion peaks at *m*/*z* 625.1765 [M+H]^+^ and 479.1184 [M+H]^+^, demonstrating the similarity with a fragmentation producing a putative ion at about *m*/*z* 317 [Quercetin+CH_3_]^+^, suggesting that two compounds belong to the quercetin backbone with an additional methoxy group. Thus, they were identified as isorhamnetin-3-robinobioside and isorhamnetin 3-*O*-glucoside, respectively. In contrast, another ion peak at *m*/*z* 579.1709 [M+H]^+^ was observed and showed the characteristic fragment of the apigenin backbone at *m*/*z* 271.0988 [Apigenin+H]^+^. Finally, this peak was assigned as apigenin-7-*O*-glucosyl-4′-*O*-rhamnoside. The identification of the above peaks detected in cluster G was accompanied by analysis of their mass fragmentation and spectral matching with previous reports on flavonoid glycosides [7,8]. Cluster O displayed the major component at *m*/*z* 355.1024 [M+H]^+^, showing the fragment of a caffeoyl moiety at *m*/*z* 163.0392 [Caffeoyl]^+^. Therefore, this peak was identified as chlorogenic acid.

This cluster also exhibited two quinic acid derivatives at *m*/*z* 517.1551 [M+H]^+^ and 517.1340 [M+H]^+^, each with an ion fragment corresponding to a caffeoyl unit of about 163 Da. Thus, these two compounds were further determined as 3,4-*O*-dicaffeoylquinic acid and 3,5-*O*-dicaffeoylquinic acid. Cluster XXXII demonstrated the protonated ion peaks at *m*/*z* 668.4373 [M+NH_4_]^+^, showing a fragment ion peak at *m*/*z* 489.3600 [M+NH_4_-Glc]^+^ due to a reduction of a glucose unit. Thus, peak 136 was identified. Furthermore, additional metabolites were identified using mass fragmentation analysis and comparison with an in-house library, as well as with entries in public mass spectrometry databases, yielding high spectral matching scores. As a result, 150 predicted compounds (Table 1) were identified from both EtOH and water extracts.

### 2.2. Identification of Antioxidants Using the LC-DPPH Method

It is important to assess the antioxidant profile of products to avoid losing their commercial and nutritional value in pharmaceutical and herbal foods. On the other hand, pharmaceuticals and food products require the development of effective, simple methods to determine the antioxidant potential of herbs specifically chosen for their active ingredients. To accomplish these objectives, integrating liquid chromatography (LC) with the DPPH radical scavenging assay is an efficient and reliable high-throughput screening approach capable of identifying antioxidant constituents while simultaneously promoting consumer health and enhancing the economic value of herbal products [9]. Firstly, the extracts interacted with DPPH under conditions that showed a reduction in the violet color observed in DPPH-treated samples compared to non-treated DPPH samples. This phenomenon may be explained by the presence of ingredients that can transfer a hydride to DPPH, forming a DPPH-H hydrazine [10]. This reaction showed a decrease in the color of the mixture, which changed from violet to pale yellow due to radical reduction. It means that EtOH and water extracts of *P. ussuriensis* may contain the active ingredient that scavenges the DPPH radical. Subsequently, the above-mentioned method was employed, demonstrating chromatographic resolution based on peak-area measurements of signal detection between treated and untreated DPPH samples under the same experimental conditions. Additionally, the above-established method was applied to develop a pre-incubation UHPLC-DPPH to determine the active components from two extracts of *P. ussuriensis*. We found that the peak areas of the detected signals in chromatograms of the treated DPPH samples differed from those of the non-treated (control) DPPH samples under the same experimental conditions (Figure 2). It may be proposed that these compounds interact with the DPPH radical in hydrazine (DPPH-H), resulting in a reduction in their peak areas in the treated DPPH chromatograms. To validate the above-established method, the strong DPPH scavenger, ascorbic acid (AA), was reacted with DPPH, and the reaction mixture was evaluated using the above LC-DPPH method. The repeatability of the DPPH, AA, and reaction mixture of AA and DPPH was determined with a precision of 0.059%, 0.032%, and 0.048% (for retention time), respectively, and 1.714%, 1.501%, and 1.752% (for peak areas), respectively, reflecting the good repeatability and precision with acceptable RSD values of the analytical method (Figure S1, Supplementary Materials). Additionally, inter- and intra-day precision were also analyzed by using the above analytical method. Results indicated that the precision values were less than 0.061% (for retention time) and 1.327% (for peak areas) on inter-day analysis (Figure S2, Supplementary Materials) as well as less than 0.049% (for retention time) and 1.406% (for peak areas) in inter-day analysis (Figure S3, Supplementary Materials). This observation reveals that the established methodology was precise and reliable for quantification. Moreover, the regression equation was established based on their reduction in peak areas compared to those of the AA sample (without DPPH addition) at the same analytical conditions (Figure S4A, Supplementary Materials). The IC_50_ values of ascorbic acid (AA) were determined as 12.15 ± 1.01 μg/mL. In contrast, the DPPH radical-scavenging activity of ascorbic acid was determined using a 96-well microplate DPPH assay, and absorbance was measured at 517 nm with a microplate reader (ELISA). Ascorbic acid showed an IC_50_ value of 10.65 ± 0.85 μg/mL.

Subsequently, the established LC-DPPH method was employed to identify the active compounds, which were highlighted with an asterisk assignment in Table 1. Among them, peaks 8–11, 17, and 21 strongly reduced the peak areas after being treated with DPPH compared to the non-treated DPPH sample (Figure 2, Table S2).

Peaks 20, 21, 25, 30, 33, 34, 38, 41, 47, 48, 61, 66, 83, 86, 110, 115, 116, 119, 123, and 124 significantly decreased peak areas when treated with DPPH compared to the control. Previous studies revealed that 8-hydroxy-6,7-dimethoxychromen-2-one (41) displayed radical-scavenging activity [11]. Tryptophan (38) also demonstrated a potent scavenger activity [12,13]. 7-hydroxycoumarin (47) and chlorogenic acid (48) showed a significant reduction in peak area between treated and untreated DPPH samples. A previous report [14] found that chlorogenic acid provides many health benefits, including mitigating oxidative stress; therefore, it may be related to adverse effects associated with radical scavenging activity and an unbalanced intracellular redox state. In particular, chlorogenic acid may mediate the free radicals generated by oxidative sources by donating hydrogen and electrons, as well as chelating metal transition ions. As a result, the free radicals were converted into a stable state via their antioxidative effect. Thus, chlorogenic acid could mitigate oxidative stress through different molecular mechanisms, as it plays key roles in cell signaling activities [15] that underlie crucial contributions [16]. Previously, 7-hydroxycoumarin showed an antioxidative effect due to its structural property. Indeed, 7-hydroxycoumarin may produce reactive coumarin radical intermediates in the presence of enzymatic catalysts, thereby mediating its prooxidant property in scavenging reactive oxidizing species. Therefore, this compound may decrease enzymatic oxidants [17] and inhibit hydroxyl, superoxide anion, and 2,2-azino-bis(3-ethylbenzothiazoline-6-sulfonic acid) (ABTS) and DPPH radicals [18]. Additionally, compound 123 exhibited strong antioxidative effects by scavenging DPPH and ABTS radicals with IC_50_ values of 4.85 ± 0.58 and 5.35 ± 0.33 μg/mL, respectively, which were remarkably higher than that of the standard BHT (IC_50_ 64.90 ± 0.75 μg/mL) [19].

Among identified compounds from EtOH extract, peaks (24, 27, 28, 30, 33, 35, 37, 40, 65, and 124) displayed a strong reduction of peak areas between treated and non-treated DPPH samples. Peaks 34, 41, 47, 61, 62, 83, 86, 111, 112, 116, 119, and 123) significantly demonstrated an antioxidative effect by reducing the peak areas between treated and non-treated DPPH samples. Peaks (22, 26, 48, 84, 115, 117, and 120) also exhibited an antioxidative effect with a weak reduction in peak areas. Among the active antioxidants detected from water extract, peaks (30, 38, 39, 41, 82, and 127) strongly reduced peak areas between treated and non-treated DPPH samples. Peaks (16–18, 22, 23, 47, 66, 80, 123, 124, 126, and 134) moderately reduced peak areas of the treated DPPH sample compared to the untreated sample. Peaks (21, 24, and 64) showed weak activity (Figures S1 and S2, Supplementary Materials). These results show that the EtOH and water extracts contain a variety of compounds that have strong antioxidant abilities, as seen by their significant decrease in DPPH peak areas. These findings demonstrate that the water and EtOH extracts contain various constituents with potent antioxidant properties, evaluated by the notable reduction in the DPPH peak regions. The discovery of these active constituents is consistent with information from previous studies demonstrating their antioxidant properties through known mechanisms and biological activity. Previously, ribitol (34) showed some antioxidative effects, in addition to its strong inhibition of cholinesterase, including acetylcholinesterase and butyrylcholinesterase [20]. Similarly, 4-caffeoylquinic acid displayed a stronger antioxidative effect than the positive control (ascorbic acid) [21]. Ibuprofen exhibited antioxidant effects by scavenging free radicals (DPPH, heme oxygenase-1 (HO), NO, and ABTS) [22]. Naringenin was reported to have some antioxidative effect [23,24] besides its novel anticancer effect in gastric carcinoma [25]. Compound 115 demonstrated significant antioxidative and hypoglycaemic effects [24]. Isorhamnetin 3-O-glucoside (116) and isorhamnetin derivatives demonstrated antioxidant capacities by inhibiting radicals, superoxide radicals, and xanthine oxidase [26]. Isorhamnetin (119) demonstrated the potential for antioxidative and anti-inflammatory activities, underlying the mechanisms of its anti-inflammatory and antioxidant effects [27]. A structural-activity relationship revealed that the antioxidative effect was negatively influenced by the glycosylation at the C-3 position of the flavonoid [28]. However, isorhamnetin and isorhamnetin 3-*O*-glucoside showed a significant antiproliferative effect towards human hepatocellular carcinoma cells (BEL-7402) and CaCO2 cells than those of flavonoid derivatives lacking a methoxy group at C-3′ of the B-ring [29]. Compound 86 showed antioxidative effect through suppressing reactive oxygen species (ROS) and nitric oxide generation (NO) by up-regulation of HO-1 via activation of nuclear factor erythroid 2-related factor 2 (Nrf2), besides its anti-inflammatory capacity by inactivation of MAPKs and the nuclear factor kappa-light-chain-enhancer of activated B cells (NF-κB) pathway resulting in decreased production of inflammatory cytokines [30]. These active constituents may enhance the overall antioxidant capacity of *P. ussuriensis*. This continuity between current screening outcomes and previous pharmacological investigations not only validates the reliability of the DPPH-guided LC approach but also highlights the therapeutic relevance of the detected active constituents.

Furthermore, the EtOH extract displayed 34 active compounds in the LC-DPPH assay, compared with 22 from the water extract (Figure S1, Supplementary Materials). The content of the active compound from EtOH is also much more than that of the water extract. However, the water extract demonstrated a stronger reduction in peak areas between treated and non-treated DPPH samples than the EtOH extract. Our data supported an overview of the discovery of active antioxidants of different extracted conditions for the development of further products on an industrial scale. To verify the antioxidative capacity of compounds, an in silico study was conducted to predict the antioxidative effect of active compounds found in the LC-DPPH assay.

### 2.3. Prediction of Active Compounds Target Antioxidative Activity

**Figure 3:**
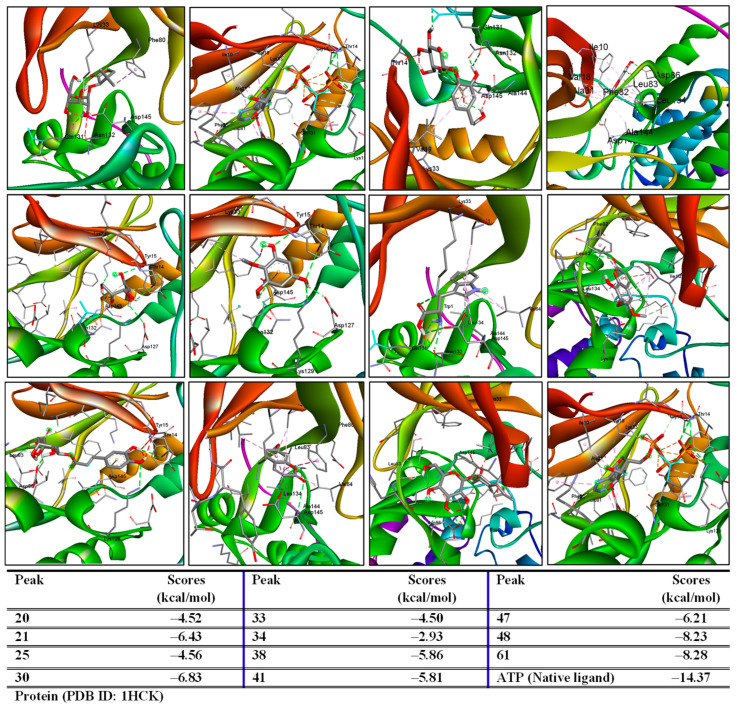
Three-dimensional interactions and binding scores of major peaks identified from EtOH extract docking into the 1HCK protein.

**Figure 4:**
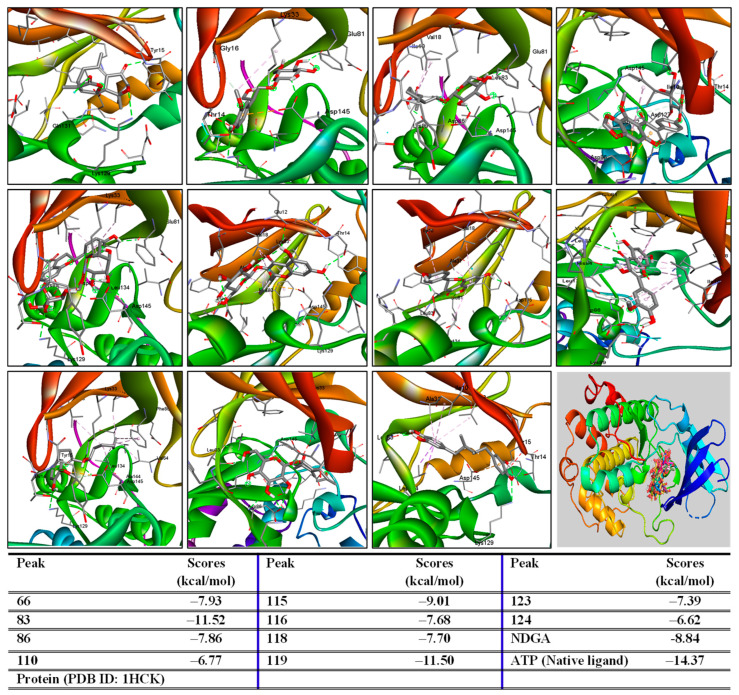
Three-dimensional interactions and binding scores of major peaks identified from water extract docking into the 1HCK protein.

Adenosine-5′-triphosphate, ATP, the native ligand, was extracted from the protein’s pdb format. The complex was then formed by redocking this ligand into the protein. The small RMSD (1.60 Å) value reflects the accuracy of the docking protocol. Thus, the major peaks of both extracts were further analyzed for their binding affinity to protein to predict their antioxidative effects. As shown in Figure 3 and Figure 4, these major active compounds and the redocked native ligand (ATP), along with the oxidant inhibitor (Nordihydroguaiaretic acid, NDGA), occupied the same region in the protein’s binding pose.

Peaks 21, 30, 38, 47, 48, 61, 66, 86, 110, 115, 116, 118, 123, and 124 were prepared as ligands docked into the 1HCK protein to establish ligand-protein complexes. They exhibited low binding energies (ΔG ranging from −11.50 to −6.21 kcal/mol). Notably, these ligands may interact with key amino acids, including THR14, TYR15, LYS30, GLU81, LEU83, LYS129, and ASP145, which were identified as important residues in the protein’s binding pose [31].

### 2.4. Target Prediction and Drug-Likeness Assessment

To assess the antioxidant activities of the above active compounds, we used the PASS online tool on the Way2Drug web server to predict their bioactivities. Data were collected by setting Pa > 0.3 (Pa represents the probability of being active) (Table 2). Compounds 20, 25, 33, 41, 47, 48, and 61 displayed moderate to high probability ranging from 44.7% to 85.5% toward antioxidant, free radical scavenging, and lipid peroxidase activities. Briefly, compound 38 showed the strongest probability (77.7%), and compounds 30, 33, 34, 41, and 47 showed moderate probability (Pa values ranging from 30.9% to 63.4%) to superoxide dismutase and chloride peroxidase activity.

Compound 38 was also predicted with a high Pa value of 86.3%, higher than the effects of compounds 33, 34, and 47 toward catalase inhibitors (Table 2). With glutathione peroxidase activity, compounds 20, 33, 34, and 38 predicted moderate activity (Pa values ranging from about 31.4–38.2%). With the nitrite reductase (NO-forming) inhibitor, compound 34 showed the highest Pa value of 76.5%, followed by compounds 30, 33, 41, and 47 with Pa values of about 34.1–54.8%. All these compounds were predicted to suppress oxidoreductase activity with Pa values ranging from 34.2% to 84.6%. Compounds 25, 30, 41, and 47 moderately suppressed NADPH oxidase activity by the PASS prediction. These observations suggested that compounds 38, 48, 25, and 34 are particularly promising as multifunctional antioxidant agents. Compound 38 stands out for enzymatic antioxidant modulation, while compounds 48 and 25 are strong radical scavengers. In silico predictions offer helpful information regarding the potential biological activities of these compounds and their potential therapeutic applications in the context of oxidative stress-related diseases.

Assessment of potential target sequences also provides insights into essential properties. We employed web server platforms to evaluate drug-likeness based on their properties of physicochemical, biochemical, pharmacokinetic, and toxicity characteristics of the above active components that possess acceptable ADME properties (Table 3). The data reflect that increasing the number of hydrogen-bond donors and acceptors is due to their direct correlation with an increase in aqueous solubility and a decrease in lipophilicity. Compounds 25 and 48 demonstrated a high number of hydrogen bonds, which proposed a difficulty in oral delivery due to poor intestinal tract absorption [32]. Except for compounds 25 and 48, all compounds possessed Lipinski’s rule of five [33]. Most of the compounds were soluble in water. Among them, compounds 21, 30, 33, 38, 41, and 47 exerted high absorption through the gastrointestinal tract; therefore, they could be promising candidates to optimize as an oral administration. Compounds 41 and 47 may cross the blood–brain barrier, suggesting that they may be impacted to reach brain penetration. Overall, the predicted ADME profiles support the potential of these compounds as orally bioavailable drug candidates, with most demonstrating favorable drug-likeness properties without significant interference with GI, BBB, and PAINS alerts, meriting further investigation.

### 2.5. Analysis of Metabolites from Both Water and EtOH Extracts

Additionally, this analytical technique was employed to estimate the content of active DPPH components and other predicted compounds from both extracts of *P. ussuriensis*. As shown in Figure S2 (Supplementary Materials), the content of compounds detected from each extract was roughly determined by calculation of their peak areas detected from the chromatograms of each extract. The phytochemical investigation of EtOH and water extracts of *P. ussuriensis* used the established method to identify predicted compounds. Both extracts detected the major components, including peaks 4, 5, 7–9, 11–14, 37, 47, 48, 61, 62, and 124. Additionally, the water extract showed four additional major peaks (2, 73, 123, and 149). The EtOH extract exhibited three major peaks, including 30, 33, 35, 115, and 118 (Figure S3 and Table S1, Supplementary Materials). Thus, the difference in composition of compounds identified from different extracts of *P. ussuriensis* indicated that the extraction method is important and affects the chemical composition of the herbal extract.

## 3. Materials and Methods

### 3.1. Plant Materials

*P. ussuriensis* fruits were obtained from Gwangyang in Fall 2022. Identification of the plant species was authorized by Prof. Mina Lee (College of Pharmacy, Sunchon National University) and Dr. Deuk-Sil Oh (Jeollanamdo Forest Research Institute). A voucher specimen of *P. ussuriensis* (SCNUP 45) was deposited in the herbarium at the Pharmacognosy laboratory (College of Pharmacy, Sunchon National University).

### 3.2. Sample Preparation

The fruits of *P. ussuriensis* were freeze-dried for 5 days using freeze-drying equipment (FDTA-5050, OFERON, Gimpo, Republic of Korea). The dried materials were divided into two batches for extraction using water and EtOH. At first, the dried materials were extracted with water at 70 °C for 3 h. Subsequently, the solution was concentrated by using a vacuum rotavator for 8 h to obtain a crude extract. On the other hand, the second batch was extracted with 100% EtOH at 70 °C for 3 h. This solution focused on a vacuum-pressure rotavator (Rotary evaporator, EYELA, Bohemia, NY, USA) to obtain the crude extract. These crude extracts were stored for further use. These extracts were dissolved in 50% high-grade MeOH and filtered through a 0.22 μm PTFE membrane (Agilent Technologies, Santa Clara, CA, USA) prior to analysis. Then, these sample solutions were analyzed using LC-MS/MS instruments.

### 3.3. LC-MS/MS Analytical Conditions

Two extracts were analyzed using an Orbitrap Exploris 120 mass spectrometer (Thermo Fisher Scientific, Sunnyvale, CA, USA) in conjunction with a Vanquish UHPLC system. The chromatograms of samples were obtained at 40 °C using a Waters column [Acquity UPLC HSS T3 (4.6 × 100 mm, 1.8 μm, Waters, Milford, MA, USA)] with an injection volume of 4 μL and a flow rate of 0.3 mL/min. Phase A, pure water with 0.1% formic acid, and phase B, ACN with 0.1% formic acid, were applied as follows to create a gradient solvent system: 3–5% (B) for 0–4 min, 5–8% (B) for 4–8 min, 8–10% (B) for 8–11 min, 10–15% (B) for 11–15 min, 15–50% (B) for 15–20 min, 50–100% (B) for 20–24 min, 100–100% (B) for 24–27 min, and 100–3% (B) for 1 min before reaching a re-equilibrium with 3% (B).

Mass evolution was set as follows: mass (*m*/*z*) range, 100–1450; spray voltage, 3500 V (positive mode); vaporizer temperature, 350 °C; ion transfer tube temperature, 325 °C; ion source gas, 50 psi; and aux gas, 10 psi. The MS/MS parameter included HCD collision energies of 15, 30, and 60 V. Full scans and data-dependent mode were selected to obtain mass spectral data. Data processing using the mzmine3.9 program and feature-based molecular network using the GNPS platform were prepared following the previous report [34].

### 3.4. Construction of Global Natural Products Social-Feature-Based Molecular Network

The molecular networking was built using the Global Natural Products Social (GNPS) web platform (https://gnps.ucsd.edu) to construct the chemical networking of extracts of *P. ussuriensis*. At first, the output file from the UHPLC-MS/MS analysis was converted to the mzXML format using the MS converter tool (https://proteowizard.sourceforge.io/). Then, the molecular network (twig job ID: b1ee54ebcb9846e4a4f6dcd1f5b6d4f8) was obtained through the GNPS web tools on the online platform. Among them, the node is the same for a single MS2 spectrum or a consensus cluster of identical MS2 spectra representing a single molecule. Each cosine score, which accounts for precursor ions, fragment ions, or peak intensities, indicates the spectral similarity between two nodes in the molecular network. The cosine score was computed based on peak intensities, precursor ions, and fragment ions. Using a cosine similarity score between nodes, a line representing related but different MS2 spectra created edges. Visualization of data obtained from the feature-based molecular network and Network annotation propagation (NAP) was conducted using Cytoscape 3.9.0 (https://cytoscape.org/).

### 3.5. Structural Annotation of Predicted Compounds

The identification of components from both extracts was performed using MS-Dial and GNPS web tools, in combination with public mass spectrometry databases. The identification of chemical IDs was mainly achieved by analyzing their isotopic patterns and mass fragmentation of mass spectra. A feature-based molecular network was visualized by using Cytoscape 3.10.

### 3.6. LC Coupled with DPPH Assay

#### 3.6.1. Reaction of Ascorbic Acid and Extracts

To screen the antioxidants derived from the extracts, ascorbic acid (AA) or each sample was prepared as follows: each sample was treated with DPPH, and the reaction mixture was incubated at 37 °C for 30 min. After a complete reaction, the sample was filtered through a 0.22 μm PTFE membrane (Agilent Technologies, Santa Clara, CA, USA) prior to further analysis. The control sample was prepared by adding the same volume of MeOH to replace the DPPH solution. Additionally, an untreated sample was prepared by either inserting only the DPPH solution or only MeOH, serving as a blank. Finally, all these samples were analyzed by using the above analytical conditions to obtain the raw data.

#### 3.6.2. Validation of the LC-DPPH Method

##### Repeatability Analysis

The strong antioxidant (ascorbic acid, AA) was selected to validate the analytical method. Ascorbic acid, DPPH, and the reaction mixture of AA and DPPH were independently prepared and run six times by using the above-established LC-MS/MS method. The retention time and peak area values were collected and analyzed to ensure precision of less than 1% (for retention time) and 2% (for peak area). The reaction mixtures of AA and DPPH at low (4 μg/mL), middle (8 μg/mL), and high (16 μg/mL) levels were analyzed for inter-day and intra-day precision. The inhibition concentration of ascorbic acid was estimated using a regression equation derived from integrating the peak areas of the reaction mixture of ascorbic acid and DPPH under the same experimental conditions.

##### Antioxidant Assay

The DPPH assay was then performed to verify the antioxidative effect of ascorbic acid using a 96-well microplate. The reaction mixture was measured for absorbance at 517 nm after incubation in the shade. The experimental procedures followed those described in our previous publication [10].

### 3.7. Molecular Docking

A molecular docking study of selected compounds was performed using a previous report [35]. Protein (1HCK) was retrieved from the Protein Data Bank (https://rcsb.org, accessed on 28 November 2025). Ligands were retrieved from PubChem (https://pubchem.ncbi.nlm.nih.gov/, accessed on 18–20 and 22 November 2025). The protein and ligand preparation were conducted using MGLTools 1.57. Molecular docking was performed using AutoDock 4.2.6. Data visualization was performed using Discovery Studio.

### 3.8. Prediction of Biological Target

The Way2Drug PASS Online server was employed to predict the biological activities of selected compounds using the default set of web servers. Thereby, the output data are consistent with the predicted activities, ranked by the probability of a chemical exhibiting activity (Pa) and inactivity (Pi). The set Pa > 0.3 was used to rank the biological activities of each compound. Each compound generated SMILES by using the ChemDraw (ver. 2016) program. Then, this SMILES string was submitted to the PASS online server for a new prediction. Subsequently, output data is produced in accordance with a set of Pa options [36].

### 3.9. Physicochemical Characteristics and Computational Approach

For each structure, SMILES were generated using ChemDraw (ver. 2016). We computed basic molecular and physicochemical descriptors using a web tool, including pharmacokinetic behavior, ADME, the number of particular atom types, molecular weight (MW), molecular refractivity (MR), and topological polar surface area (TPSA). Based on the average values of the various computational factors, lipophilicity was evaluated using five alternative prediction models (XLOGP, WLOGP, MLOGP, SILICOS-IT, and iLOGP) and a consensus logP estimation. Similarly, three different models were used to determine the solubility in water. The goal of the ADME/pharmacokinetics analysis was to estimate key parameters, including gastrointestinal absorption, solubility, and blood–brain barrier (BBB) permeability. To achieve accurate predictions, the SwissADME tool [37] leverages a support vector machine (SVM) algorithm. This SVM is trained on comprehensive, rigorous datasets that differentiate between known inhibitors and non-inhibitors, and between substrates and non-substrates.

### 3.10. Drug Likeness Prediction

The drug-likeness analysis was carried out using the validated rules used as high-throughput screening filters, as follows: Lipinski (Pfizer, New York, NY, USA), Ghose (Amgen, Thousand Oaks, CA, USA), Veber (GSK, London, UK), Egan (Pharmacia, Peapack, NJ, USA), and Muegge (Bayer, Leverkusen, Germany). The Abbott bioavailability score was calculated to predict the probability of a 10% oral bioavailability. These filters have been developed to assess drug-likeness, i.e., to predict whether a chemical entity is likely to have useful pharmacokinetic properties, based on therapeutic principles and parameters such as molecular weight, LogP, and numbers of hydrogen bond acceptors and donors [9].

### 3.11. Statistical Analysis

Data were represented as the means ± standard deviations (S.D.) (*n* ≥ 3) of at least three replicates. The nonparametric one-way ANOVA followed by Dunnett’s multiple-comparison test was performed using GraphPad Prism version 8.0.1 (GraphPad Software, La Jolla, CA, USA) for statistical analyses.

## 4. Conclusions

In conclusion, Pear is a high-value crop and is popular in many parts of the world. Our study has developed an analytical method using high-tech equipment, a technology increasingly popular in pharmaceutical technology. The application and combination with supporting tools have brought high efficiency and utility in analyzing and determining the chemical composition of pear extract samples here. The detected chemical components span a diverse range of substance classes, reflecting the complex metabolic processes within *P. ussuriensis*. The application of advanced technologies not only helps orient well but also improves the optimization of input materials, specifically by identifying substances with antioxidant activity while consuming less input materials when using the above analytical method combined with DPPH to target antioxidants. This approach has helped identify active ingredients and evaluate their composition, as well as the other chemical components present in each extract from this plant. Therefore, the results obtained highlight the value of *P. ussuriensis* and guide the development of antioxidant therapeutic products from its active constituents.

## Figures and Tables

**Table 1:** Identification of components from *P. ussuriensis* extracts.

No.	Compound	RT (min)	Formula	Adduct	*m*/*z* (Da)	Error (mDa)
1	Unknown	0.665	-	[M+H]^+^	241.9996	-
2	Unknown	0.708	-	[M+H]^+^	219.0265	-
3	Unknown	0.739	-	[M+H]^+^	320.8663	-
4	Mannitol	0.812	C_9_H_10_O_5_	[M+K]^+^	221.0421	0.1220
5	L-Proline	0.877	C_5_H_9_NO_2_	[M+H]^+^	116.0706	0.0230
6	Sucrose	0.887	C_12_H_22_O_11_	[M+H-H_2_O]^+^	325.1130	0.7020
7	(*Z*)-2-methyl-4-[(2*R*,3*R*,4*S*,5*S*,6*R*)-3,4,5-trihydroxy-6-(hydroxymethyl)oxan-2-yl]oxybut-2-enenitrile	0.940	C_11_H_17_NO_6_	[M+H]^+^	260.1128	0.0000
8	2-(5,6-Dihydroxy-3-methoxycarbonylcyclohex-3-en-1-yl)oxypropanoic acid	0.940	C_11_H_16_O_7_	[M+NH_4_]^+^	278.1234	0.0000
9	Mycosporine serinol	1.063	C_11_H_19_NO_6_	[M+H]^+^	262.1285	0.5190
10	Unknown	1.235	C_12_H_20_N_2_O_3_	[M+H]^+^	241.1547	0.0059
11	Adenosine	1.235	C_10_H_13_N_5_O_4_	[M+H]^+^	268.1040	0.0000
12	Unknown	1.303	-	[M+H]^+^	276.1442	-
13	Unknown	1.303	-	[M+H]^+^	294.1548	-
14	Norleucine	1.313	C_6_H_13_NO_2_	[M+H]^+^	132.1019	0.0150
15	Kinetin riboside	1.559	C_15_H_17_N_5_O_5_	[M+H]^+^	348.1290	1.2820
16	(2*aS*,5*aR*,7*R*,8*aR*)-6-Hydroxy-6-[2-(2-hydroxy-5-oxo-2,5-dihydro-3-furanyl)ethyl]-2*a*,5*a*,7-trimethyldecahydro-2*H*-naphtho [1,8-bc]furan-2-one *	2.093	C_20_H_28_O_6_	[M+H]^+^	365.1919	3.9670
17	Picraquassioside D *	2.120	C_13_H_18_O_8_	[M+Na]^+^	325.0895	0.0920
18	Benzyl glucopyranoside	2.337	C_12_H_20_O_8_	[M+H+ CH_3_CHO]^+^	315.1051	2.8990
19	Benzanthrone	2.347	C_17_H_10_O	[M+H]^+^	231.0839	3.5100
20	(3*R*)-4,4-Dimethyl-2-oxotetrahydro-3-furanyl *β*-D-glucopyranoside *	2.468	C_12_H_20_O_8_	[M+Na]^+^	315.1051	0.0610
21	Picrotin *	2.524	C_15_H_18_O_7_	[M+H]^+^	311.1126	0.0920
22	Riboflavin *	2.674	C_17_H_20_N_4_O_6_	[M+Na]^+^	399.1262	0.0920
23	Saccatoside	3.613	C_30_H_38_O_16_	[M+H]^+^	655.2211	2.1360
24	Unknown *	3.630	-	[M+H]^+^	392.0953	-
25	2-Hydroxy-4-(2-hydroxyethyl)phenyl *β*-D-glucopyranoside *	3.655	C_14_H_20_O_8_	[M+Na]^+^	339.1051	0.0920
26	Dianthoside *	3.692	C_12_H_16_O_8_	[M+H]^+^	289.0894	2.3800
27	Pantothenic acid *	4.019	-	[M+H]^+^	220.1179	0.0150
28	Methocarbamol *	4.019	C_11_H_15_NO_5_	[M+H]^+^	242.0999	2.4110
29	5,7-Dihydroxy-2-methyl-8-[(2*S*,3*R*,4*R*,5*S*,6*R*)-3,4,5-trihydroxy-6-(hydroxymethyl)oxan-2-yl]chromen-4-one	4.292	C_16_H_18_O_9_	[M+H]^+^	355.1000	2.3500
30	Ellagic acid *	4.465	C_14_H_6_O_8_	[M+H]^+^	303.1051	0.8850
31	4-(Hexopyranosyloxy)-3-methoxybenzoic acid *	4.544	C_14_H_18_O_9_	[M+Na]^+^	353.0844	0.0920
32	(2*R*,3*S*,4*S*,5*R*,6*R*)-2-[[(2*R*,3*R*,4*R*)-3,4-dihydroxy-4-(hydroxymethyl)oxolan-2-yl]oxymethyl]-6-[2-(4-hydroxyphenyl)ethoxy]oxane-3,4,5-triol	4.725	C_19_H_28_O_11_	[M+H]^+^	433.1680	2.4410
33	Iretol *	5.641	C_7_H_8_O_4_	[M+H]^+^	157.0495	0.0000
34	Ribitol *	5.641	C_5_H_12_O_5_	[M+Na]^+^	175.0601	0.1070
35	Unknown *	5.669	-	[M+H]^+^	243.0474	-
36	Geniposidic Acid	5.778	C_16_H_22_O_10_	[M+Na]^+^	397.1105	0.0310
37	Unknown *	5.896	C_11_H_9_NO_2_	[M+H]^+^	188.0706	-
38	Tryptophan *	5.917	C_11_H_12_N_2_O_2_	[M+H]^+^	205.0971	0.0460
39	2-(1,2-Dihydroxyethyl)-3-[(*E*)-3-(3,4-dihydroxyphenyl)prop-2-enoyl]oxy-4-hydroxy-3,4-dihydro-2*H*-pyran-6-carboxylic acid *	6.429	C_17_H_18_O_10_	[M+H]^+^	383.0949	2.3190
40	Secologanol *	6.696	C_17_H_26_O_10_	[M+Na]^+^	413.1418	0.0000
41	8-Hydroxy-6,7-dimethoxychromen-2-one *	7.212	C_11_H_10_O_5_	[M+H]^+^	223.0577	2.3960
42	Methylxanthoxylin	7.884	C_11_H_14_O_4_	[M+H]^+^	211.0965	0.5040
43	Hydrastine	8.545	C_21_H_21_NO_6_	[M+H]^+^	406.1344	4.6690
44	3,4-Di-*O*-caffeoylquinic acid	9.348	C_25_H_24_O_12_	[M+H]^+^	517.1551	0.0211
45	Unknown	9.646	-	[M+H]^+^	319.1514	-
46	Unknown	9.677	-	[M+H]^+^	660.1710	-
47	7-Hydroxy-coumarin *	10.170	C_9_H_6_O_3_	[M+H]^+^	163.03894	0.0310
48	Chlorogenic acid *	10.187	C_16_H_18_O_9_	[M+H]^+^	355.1024	0.0014
49	Grandidentoside	10.802	C_21_H_28_O_10_	[M+Na]^+^	463.1575	0.0000
50	Periplanetin	11.000	C_13_H_16_O_7_	[M+Na]^+^	307.0789	0.0610
51	Junipediol B 8-*O*-glucoside	11.086	C_16_H_22_O_9_	[M+Na]^+^	381.1157	0.0920
52	Chicoric acid	11.203	C_22_H_18_O_12_	[M+H]^+^	163.0390	0.0526
53	7-Hydroxy-5-methyl-2-(2-oxopropyl)-8-[3,4,5-trihydroxy-6-(hydroxymethyl)oxan-2-yl]chromen-4-one	11.395	C_19_H_22_O_9_	[M+H]^+^	395.1313	2.3800
54	Syringin	11.400	C_17_H_24_O_9_	[M+NH_4_]^+^	390.1759	0.0610
55	Cyclopenta[c]pyran-4-carboxylic acid, 1-(*β*-D-glucopyranosyloxy)-1,4*a*,5,6,7,7*a*-hexahydro-4*a*-hydroxy-7-methyl-5-oxo-, 2-(3,4-dihydroxyphenyl)ethyl ester, (1*S*,4*aR*,7*aR*)-	11.612	C_24_H_30_O_13_	[M+NH_4_]^+^	544.2026	0.1220
56	(2*S*,3*R*,4*S*,5*S*,6*R*)-2-[[(1*S*,4*aR*,7*aS*)-7-(hydroxymethyl)-1,4*a*,5,7*a*-tetrahydrocyclopenta[c]pyran-1-yl]oxy]-6-(hydroxymethyl)oxane-3,4,5-triol	12.368	C_15_H_22_O_8_	[M+H]^+^	331.1364	2.3190
57	Procyanidin B2	12.469	C_30_H_26_O_12_	[M+H]^+^	579.1498	0.1220
58	Cichorioside B	12.564	C_21_H_28_O_10_	[M+H]^+^	441.1731	2.4410
59	(+)-Genipin	13.480	C_11_H_14_O_5_	[M+H]^+^	227.0914	0.0150
60	(−)-Epicatechin	13.720	C_15_H_14_O_6_	[M+H]^+^	291.0863	0.0003
61	4-[4-(*β*-D-glucopyranosyloxy)-2-hydroxy-2,6,6-trimethylcyclohexylidene]-3-buten-2-one *	13.934	C_19_H_30_O_8_	[M+Na]^+^	409.1833	0.0610
62	Unknown *	13.937	-	[M+H]^+^	207.1379	-
63	Roseoside	13.937	C_19_H_30_O_8_	[M+H]^+^	387.2014	0.0610
64	Cinnamoside *	14.433	C_24_H_38_O_12_	[M+Na]^+^	541.2256	0.0610
65	Unknown *	14.476	-	[M+H]^+^	247.0812	-
66	9,14-Dimethyl-5-methylidene-3,13-dioxatetracyclo [8.4.0.02,6.012,14]tetradec-9-ene-4,11-dione *	14.481	C_15_H_16_O_4_	[M+H]^+^	261.1121	0.0000
67	(+)-Lyoniresinol 9-glucoside	14.815	C_28_H_38_O_13_	[M+NH_4_]^+^	600.2651	0.0610
68	Unknown	14.881	-	[M+H]^+^	388.2540	-
69	Icariside B8	14.952	C_19_H_32_O_8_	[M+Na]^+^	411.1990	0.0310
70	(*E*)-3-[4-methoxy-2-[(2*S*,3*R*,4*S*,5*S*,6*R*)-3,4,5-trihydroxy-6-(hydroxymethyl)oxan-2-yl]oxyphenyl]prop-2-enoic acid	15.903	C_16_H_20_O_9_	[M+H]^+^	357.1157	2.3190
71	Unknown	15.908	-	[M+H]^+^	352.1603	-
72	3,4-Dihydroxyallylbenzene 3,4-di-*O*-glucoside	15.967	C_21_H_30_O_12_	[M+NH_4_]^+^	492.2076	0.0000
73	Unknown	16.109	-	[M+H]^+^	343.1364	-
74	Acarbose	16.109	C_25_H_43_NO_18_	[M+NH_4_]^+^	663.2842	2.4410
75	5,7-Dihydroxy-2-[3-hydroxy-4-[(2*S*,3*R*,4*S*,5*S*,6*R*)-3,4,5-trihydroxy-6-(hydroxymethyl)oxan-2-yl]oxyphenyl]-3-[(2*S*,3*R*,4*S*,5*S*,6*R*)-3,4,5-trihydroxy-6-(hydroxymethyl)oxan-2-yl]oxychromen-4-one	16.416	C_27_H_30_O_17_	[M+H]^+^	627.1557	0.0007
76	3-[(2*S*,3*R*,4*S*,5*S*,6*R*)-6-[[(2*R*,3*R*,4*R*,5*R*,6*S*)-3-[(2*S*,3*R*,4*R*)-3,4-dihydroxy-4-(hydroxymethyl)oxolan-2-yl]oxy-4,5-dihydroxy-6-methyloxan-2-yl]oxymethyl]-3,4,5-trihydroxyoxan-2-yl]oxy-2-(3,4-dihydroxyphenyl)-5,7-dihydroxychromen-4-one	16.762	C_32_H_38_O_20_	[M+H]^+^	743.2035	0.0034
77	Unknown	16.829	-	[M+H]^+^	205.1434	-
78	Gallomyrtucommulone C	16.998	C_27_H_36_O_13_	[M+NH_4_]^+^	586.2496	0.2440
79	2-(3,4-Dihydroxyphenyl)-5,7-dihydroxy-3-[(2*S*,3*R*,4*S*,5*S*,6*R*)-3,4,5-trihydroxy-6-[[(2*S*,3*R*,4*S*,5*R*)-3,4,5-trihydroxyoxan-2-yl]oxymethyl]oxan-2-yl]oxychromen-4-one	17.120	C_26_H_28_O_16_	[M+H]^+^	597.1452	0.0002
80	Unknown *	17.276	-	[M+H]^+^	446.1656	-
81	Rhodioloside *	17.296	C_14_H_20_O_7_	[M+H]^+^	301.1258	2.3500
82	Unknown *	17.316	-	[M+H]^+^	542.2596	-
83	Junipediol A *	17.323	C_16_H_24_O_9_	[M+Na]^+^	383.1314	0.0920
84	5-Hydroxy-2-[2-hydroxy-3-[(2*S*,3*R*,4*S*,5*S*,6*R*)-3,4,5-trihydroxy-6-(hydroxymethyl)oxan-2-yl]oxyphenyl]-7,8-dimethoxychromen-4-one *	17.323	C_23_H_24_O_12_	[M+Na]^+^	515.1185	2.5020
85	Guaiphenesin *	17.333	C_10_H_14_O_4_	[M+H]^+^	199.0965	0.0460
86	7-Methyl-1-[(2*S*,3*R*,4*S*,5*S*,6*R*)-3,4,5-trihydroxy-6-(hydroxymethyl)oxan-2-yl]oxy-1,4*a*,5,6,7,7*a*-hexahydrocyclopenta[c]pyran-4-carboxylic acid *	17.333	C_16_H_24_O_9_	[M+NH_4_]^+^	378.1759	0.0610
87	Fumitremorgin A	17.350	C_32_H_41_N_3_O_7_	[M+Na]^+^	602.2811	2.6250
88	Ouabain	17.350	C_29_H_44_O_12_	[M+Na]^+^	607.2364	1.9530
89	8-Hydroxypinoresinol-4′-*O*-*β*-D-Glucopyranoside	17.489	C_26_H_32_O_12_	[M+NH_4_]^+^	554.2233	0.0000
90	1,2,3-Trimethoxy-5-[2-[4-(3-methylbut-2-enoxy)phenyl]ethyl]benzene	17.529	C_22_H_28_O_4_	[M+Na]^+^	379.1865	1.4950
91	Unknown	17.535	-	[M+H]^+^	520.3329	-
92	Rutin	17.593	C_27_H_30_O_16_	[M+H]^+^	611.1616	0.9770
93	7-Hydroxy-2-(4-hydroxyphenyl)-8-[(2*S*,3*R*,4*R*,5*S*,6*R*)-3,4,5-trihydroxy-6-(hydroxymethyl)oxan-2-yl]chromen-4-one	17.619	C_21_H_20_O_9_	[M+H]^+^	417.1157	2.3190
94	Clerodin	17.634	C_24_H_34_O_7_	[M+Na]^+^	457.2408	1.2510
95	Quercetin	17.659	C_15_H_10_O_7_	[M+H]^+^	303.0499	0.0310
96	Unknown	17.673	-	[M+H]^+^	554.2708	-
97	Unknown	17.708	-	[M+H]^+^	329.1596	-
98	Hyperoside	17.767	C_21_H_20_O_12_	[M+H]^+^	465.1028	0.0000
99	4-[3-[(2*S*,3*R*,4*S*,5*S*,6*R*)-6-[[(2*R*,3*R*,4*R*)-3,4-dihydroxy-4-(hydroxymethyl)oxolan-2-yl]oxymethyl]-3,4,5-trihydroxyoxan-2-yl]oxy-2-hydroxy-3-methylbutoxy]furo [3,2-g]chromen-7-one	17.787	C_27_H_34_O_15_	[M+H]^+^	599.1973	0.2440
100	Asperulosidic acid	17.792	C_18_H_24_O_12_	[M+H-H_2_O]^+^	415.1235	0.0000
101	Protopine	17.827	C_20_H_19_NO_5_	[M+H]^+^	354.1760	1.6780
102	Unknown	17.906	-	[M+H]^+^	455.2252	-
103	2-Phenylethyl 3-*O*-(4-carboxy-3-hydroxy-3-methylbutanoyl)-*β*-D-glucopyranoside	17.946	C_20_H_28_O_10_	[M+H]^+^	429.1732	2.3800
104	(*E*)-Coniferin	18.018	C_16_H_22_O_8_	[M+Na]^+^	365.1208	0.0610
105	3-[[(2*R*,3*S*,4*S*,5*R*,6*S*)-6-[2-(3,4-Dihydroxyphenyl)-5,7-dihydroxy-4-oxochromen-3-yl]oxy-3,4,5-trihydroxyoxan-2-yl]methoxy]-3-oxopropanoic acid	18.058	C_24_H_22_O_15_	[M+H]^+^	551.1032	0.0610
106	[(1*R*,2*E*,8*S*,10*R*,11*S*)-10,11-Dihydroxy-6-(methoxymethyl)-1,10-dimethyl-5-oxo-4,14-dioxatricyclo [9.2.1.03,7]tetradeca-2,6-dien-8-yl] 2-methylprop-2-enoate	18.102	C_20_H_26_O_8_	[M+H]^+^	395.1677	1.9840
107	Isorhamnetin-3-*O*-galactoside-6-*O*-rhamnoside	18.156	C_28_H_32_O_16_	[M+H]^+^	625.1765	0.1830
108	Unknown	18.195	-	[M+H]^+^	343.2092	-
109	9-Methoxy-7-[4-[(2*S*,3*R*,4*S*,5*S*,6*R*)-3,4,5-trihydroxy-6-(hydroxymethyl)oxan-2-yl]oxyphenyl]-[1,3]dioxolo [4,5-g]chromen-8-one *	18.195	C_23_H_22_O_11_	[M+Na]^+^	497.1079	2.5020
110	5-Hydroxy-7-[3,4,5-trihydroxy-6-(hydroxymethyl)oxan-2-yl]oxy-2-[4-(3,4,5-trihydroxy-6-methyloxan-2-yl)oxyphenyl]chromen-4-one	18.221	C_27_H_30_O_14_	[M+H]^+^	579.1709	0.0001
111	(3*R*,5*R*)-3,5-*Bis*[[(*E*)-3-(3,4-dihydroxyphenyl)prop-2-enoyl]oxy]-1,4-dihydroxycyclohexane-1-carboxylic acid	18.245	C_25_H_24_O_12_	[M+H]^+^	517.1340	0.0018
112	3,5-Dicaffeoylquinic acid	18.250	C_25_H_24_O_12_	[M+H-H_2_O]^+^	499.1236	0.1220
113	Methyl (2*S*,4*aS*,6*aR*,7*R*,10*aR*,10*bR*)-2-(3-furanyl)-1,4,4*a*,5,6,6*a*,7,10,10*a*,10*b*-decahydro-7-hydroxy-6*a*,10*b*-dimethyl-4-oxo-2*H*-naphtho [2,1-c]pyran-7-carboxylate *	18.225	C_21_H_26_O_6_	[M+Na]^+^	397.1646	2.4720
114	5-Hydroxy-2-(4-hydroxyphenyl)-7-[(2*S*,3*R*,4*S*,5*S*,6*R*)-3,4,5-trihydroxy-6-[[(2*R*,3*R*,4*R*,5*R*,6*S*)-3,4,5-trihydroxy-6-methyloxan-2-yl]oxymethyl]oxan-2-yl]oxy-2,3-dihydrochromen-4-one *	18.275	C_27_H_32_O_14_	[M+H]^+^	581.1866	0.1830
115	Isorhamnetin 3-*O*-glucoside *	18.285	C_22_H_22_O_12_	[M+H]^+^	479.1184	0.0024
116	(2*S*,3*R*,4*S*,5*S*,6*R*)-2-[4-[(3*S*,3*aR*,6*S*,6*aR*)-3-(4-hydroxy-3,5-dimethoxyphenyl)-1,3,3*a*,4,6,6*a*-hexahydrofuro [3,4-c]furan-6-yl]-2,6-dimethoxyphenoxy]-6-(hydroxymethyl)oxane-3,4,5-triol	18.314	C_28_H_36_O_13_	[M+NH_4_]^+^	598.2496	0.1830
117	(+)-Syringaresinol *β*-D-glucoside	18.314	C_28_H_36_O_13_	[M+NH_4_]^+^	598.2496	0.1830
118	Naringenin *	18.320	C_15_H_12_O_5_	[M+H]^+^	273.0757	0.0920
119	Isorhamnetin *	18.364	C_16_H_12_O_7_	[M+H]^+^	317.0657	0.0043
120	Unknown	18.416	-	[M+H]^+^	476.2128	-
121	(2*E*,4*E*,8*E*)-7,13-Dihydroxy-4,8,12-trimethyltetradeca-2,4,8-trienoic acid	18.440	C_17_H_28_O_4_	[M+H]^+^	297.2038	2.2580
122	Cyclopentaneacetic acid	18.555	C_18_H_30_O_8_	[M+Na]^+^	397.1834	0.0920
123	(5*R*)-5-Hydroxy-1-(4-hydroxy-3-methoxyphenyl)decan-3-one *	18.691	C_17_H_26_O_4_	[M+H]^+^	295.1881	2.3500
124	(4*S*,4*aR*)-4-(Hydroxymethyl)-3,4*a*,8,8-tetramethyl-4*a*,5,6,7,8,8*a*-hexahydro-1(4*H*)-naphthalenone *	18.695	C_15_H_24_O_2_	[M+H]^+^	237.1849	0.0150
125	9-Hydroxyageraphorone	18.696	C_15_H_24_O_2_	[M+H-H_2_O]^+^	219.1744	0.0000
126	Acantrifoside E *	18.856	C_17_H_24_O_8_	[M+Na]^+^	379.1364	0.0610
127	6-Hydroxy-2,6,10,10-tetramethyl-1-oxaspiro [4.5]dec-8-yl 6-*O*-[(2*R*,3*R*,4*R*)-3,4-dihydroxy-4-(hydroxymethyl)tetrahydro-2-furanyl]-*β*-D-glucopyranoside*	18.856	C_24_H_42_O_12_	[M+H-H_2_O]^+^	505.2658	1.4950
128	Unknown	18.912	-	[M+H]^+^	764.3127	-
129	5,5,8*a*-Trimethyl-1,4,4*a*,5,6,7,8,8*a*-octahydronaphthalene-1,2-dicarbaldehyde	18.937	C_15_H_22_O_2_	[M+H+ CH_3_COOH]^+^	293.1723	0.6710
130	(1*β*,2*α*,9*ξ*,11*β*,12*α*,15*β*)-1,2,11,12,14,15-hexahydroxy-Picras-4-en-16-one	19.017	C_20_H_30_O_8_	[M+H]^+^	399.1990	2.3500
131	Unknown	19.107	-	[M+H]^+^	439.2303	-
132	2-{[6-*O*-(6-Deoxy-*α*-L-mannopyranosyl)-*β*-D-glucopyranosyl]oxy}-3-methylbutanenitrile	19.385	C_17_H_29_NO_10_	[M+Na]^+^	430.1708	2.4410
133	4-[(*E*)-3-[(2*R*,3*R*,4*S*,5*S*,6*R*)-3-[(2*S*,3*R*,4*R*)-3,4-Dihydroxy-4-(hydroxymethyl)oxolan-2-yl]oxy-4,5-dihydroxy-6-(hydroxymethyl)oxan-2-yl]oxybut-1-enyl]-3,5,5-trimethylcyclohex-2-en-1-one	19.478	C_24_H_38_O_11_	[M+NH_4_]^+^	520.2753	0.0610
134	7-Hydroxy-3-[4-hydroxy-3-(3-methylbut-2-enyl)phenyl]chromen-4-one *	19.942	C_20_H_18_O_4_	[2M+Na]^+^	667.2367	6.7140
135	Unknown	20.137	-	[M+H]^+^	607.2153	-
136	1-*O*-[(2*α*,3*β*,5*ξ*,9*ξ*,18*ξ*)-2,3,19-trihydroxy-28-oxours-12-en-28-yl]- *β*-D-Glucopyranose	20.255	C_36_H_58_O_10_	[M+NH_4_]^+^	668.4373	0.0003
137	Berberine	20.441	C_20_H_18_NO_4_	[M+H]^+^	337.1984	3.9060
138	Unknown	20.572	-	[M+H]^+^	226.1801	-
139	Parthenolide	20.572	C_15_H_20_O_3_	[M+NH_4_]^+^	266.1726	2.3800
140	(10*E*,12*E*)-9-Hydroxyoctadeca-10,12-dienoic acid	20.602	C_18_H_32_O_3_	[M+H-H_2_O]^+^	279.2319	0.0610
141	8-Hydroxy-8-(3-octyloxiran-2-yl)octanoic acid	20.602	C_18_H_34_O_4_	[M+H-H_2_O]^+^	297.2425	0.0610
142	Unknown	20.602	-	[M+H]^+^	355.2456	-
143	Unknown	20.644	-	[M+H]^+^	555.2203	-
144	Phytosphingosine	21.989	C_18_H_39_NO_3_	[M+H]^+^	318.3003	0.0310
145	Unknown	22.186	-	[M+H]^+^	316.3211	-
146	Tormentic acid	22.661	C_30_H_48_O_5_	[M+H]^+^	489.3574	0.0004
147	(2*α*,3*β*,5*ξ*,9*ξ*,19*α*)-2,3,19-trihydroxy-olean-12-en-28-oic ξacid	22.672	C_30_H_48_O_5_	[M+H-H_2_O]^+^	471.3469	0.0009
148	Unknown	23.887	-	[M+H]^+^	452.3218	-
149	5,7-Dihydroxy-6-[3,4,5-trihydroxy-6-(hydroxymethyl)oxan-2-yl]-8-[3,4,5-trihydroxy-6-(hydroxymethyl)oxan-2-yl]oxychromen-2-one	25.445	C_21_H_26_O_15_	[M+NH_4_]^+^	536.1656	4.5780
150	Unknown	27.406	-	[M+H]^+^	612.3722	-

* Compound has an antioxidant activity, evaluated by the LC-DPPH assay. *-* Not identified.

**Table 2:** Biological activities of compounds derived from the PASS online tool.

Compound	Antioxidant	Free Radical Scavenger	Lipid Peroxidase Inhibitor	Superoxide Dismutase Inhibitor	Catalase Inhibitor	Glutathione Peroxidase Inhibitor	Nitrite Reductase (NO-forming) Inhibitor	Oxidoreductase Inhibitor	Chloride Peroxidase Inhibitor	NADPH Oxidase Inhibitor
20	62.8	60.1	52.1	-	-	36.3	-	69.6	-	-
21	-	-	-	-	-	-	-	69.6	-	-
25	69.0	78.3	78.9	-	-	-	-	70.6	-	32.7
30	69.9	59.6	53.6	42.3	30.9	26.0	53.2	62.4	53.7	32.4
33	54.7	56.3	60.6	50.5	32.8	31.4	54.8	60.8	63.4	-
34	-	-	-	63.0	57.0	38.2	76.5	35.9	63.4	-
38	-	-	-	77.7	86.3	35.9	-	67.4	33.3	-
41	56.2	73.9	62.3	37.4	-	-	34.1	75.5	35.2	36.2
47	56.3	67.1	57.0	40.7	45.9	-	54.3	83.4	50.4	36.2
48	78.5	85.6	85.5	-	-	-	-	84.6	-	-
61	69.9	81.0	44.7	-	-	-	-	59.2	-	-

Data was obtained from https://www.way2drug.com/passonline/predict, accessed on 20 November 2025. Data expressed for Pa values (%) which were the probability of activation for consideration using the threshold of Pa > 30%. -: Non-prediction.

**Table 3:** ADME and Drug-likeness properties of the active compounds.

Compound	MW	Lipophilicity	TPSA	H Donors	H Acceptors	Solubility	GI	BBB	Bioavailability	Lipinski	Pain
20	292.1158	−1.02	125.68	4	8	Soluble	-	-	0.55	+	-
21	310.1053	0.11	105.59	2	7	Soluble	+	-	0.55	+	-
25	316.1158	−0.95	139.84	6	8	Soluble	-	-	0.55	-	-
30	302.1051	1.00	141.34	4	8	Soluble	+	-	0.55	+	-
33	156.0423	0.63	69.92	3	4	Soluble	+	-	0.55	+	-
34	152.0685	−1.80	101.15	5	5	Soluble	-	-	0.55	+	-
38	204.0899	0.18	79.11	3	3	Soluble	+	-	0.55	+	-
41	222.0528	1.49	68.90	1	5	Soluble	+	+	0.55	+	-
47	162.0317	1.51	50.44	1	3	Soluble	+	+	0.55	+	-
48	354.0951	−0.39	164.75	6	9	Soluble	-	-	0.11	-	+
61	386.1941	−0.14	136.68	5	8	Soluble	-	-	0.55	+	-

Data were obtained from the Swiss target prediction web server. +: Pass. -: Not Pass.

## Data Availability

The original contributions presented in this study are included in the article/Supplementary Materials. Further inquiries can be directed to the corresponding author.

## Supplementary Materials

The following supporting information can be downloaded at https://www.mdpi.com/article/10.3390/ph19010192/s1. Figure S1: Analyzing the repeatability of DPPH, AA, and AA+DPPH reaction mixture by using the established LC-DPPH method. Rt represents the retention time (min). PA describes the peak areas detected from the chromatograms; Figure S2: Inter-day precision of AA+DPPH reaction mixture at different concentrations analyzed by applying the established LC-DPPH method. Rt represents the retention time (min). PA describes the peak areas detected from the chromatograms; Figure S3: Intra-day precision of AA+DPPH reaction mixture at different concentrations analyzed by applying the established LC-DPPH method. Rt represents the retention time (min). PA describes the peak areas detected from the chromatograms; Figure S4: Regress equation of ascorbic acid determined by LC-DPPH (A) and Elisa assay (B); Figure S5: Composition (A) and reduction (%, B) of peak areas of active DPPH components from EtOH extract; Figure S6: Composition (A) and reduction (%, B) of peak areas of active DPPH components from water extract; Figure S7: Peak areas of compounds identified from *P. ussuriensis* extracts. Table S1: Identification of components from SDB fractions. Table S2: Spectra of identified compound has an antioxidant activity, evaluated by LC-DPPH.
