# Supplementary material for: Identification of Metabolites and Antioxidant Constituents from Pyrus ussuriensis
Source: Pharmaceuticals (Basel). 2026 Jan 22;19(1):192. doi: 10.3390/ph19010192 (PMC12844793; doi:10.3390/ph19010192)
Supplement: Supplementary file 1 [file pharmaceuticals-19-00192-s001.zip › pharmaceuticals-4055720-supplementary.pdf]

pharmaceuticals

MDPI

Article

Identification of Metabolites and Antioxidant Constituents from *Pyrus ussuriensis*

Ducdat Le, Thientam Dinh, Soojung Yu, Yun-Jin Lim, Hae-In Lee, Jin Woo Park, Deuk-Sil Oh and Mina Lee

DPPH (0.4 mg/mL)	No	Rt	PA
Run	1	24.85	3418802.72
	2	24.87	3467296.03
	3	24.88	3546652.64
	4	24.86	3563461.38
	5	24.86	3537579.67
	6	24.89	3567982.46
Average		24.86833333	3516962.483
SD		0.014719601	60289.0619
%RSD		0.059190141	1.714236708

AA 8 (ppm)	No	Rt	PA
Run	1	25.4	239741.31
	2	25.41	230543.97
	3	25.39	232712.59
	4	25.39	233330.68
	5	25.4	233587.77
	6	25.39	229860.36
Average		25.39666667	233296.1133
SD		0.008164966	3502.742123
%RSD		0.032149754	1.501414693

AA + DPPH (8 ppm)	No	Rt	PA
Run	1	24.85	3092749.83
	2	24.87	3039640.75
	3	24.88	3033039.45
	4	24.86	3105357.31
	5	24.85	3182187.41
	6	24.87	3104472.99
Average		24.86333333	3092907.957
SD		0.012110601	54187.11209
%RSD		0.04870868	1.751979459

Figure S1. Analyzing the repeatability of DPPH, AA, and AA+DPPH reaction mixture by using the established LC-DPPH method. Rt represents the retention time (min). PA describes the peak areas detected from the chromatograms.

AA + DPPH (4 ppm)	No	Rt	PA
Run	1	24.88	3409639.17
	2	24.89	3408250.74
	3	24.87	3369380.78
Mean		24.88	3395756.897
SD		0.01	22852.93378
%RSD		0.040192926	0.672984977

AA + DPPH (8 ppm)	No	Rt	PA
Run	1	24.85	3093695.8
	2	24.87	3038366.55
	3	24.88	3014869.5
Mean		24.86666667	3048977.283
SD		0.015275252	40470.20063
%RSD		0.061428629	1.327336903

AA + DPPH (16 ppm)	No	Rt	PA
Run	1	24.87	2401484.73
	2	24.89	2410639.81
	3	24.87	2419377.93
Mean		24.87666667	2410500.823
SD		0.011547005	8947.409655
%RSD		0.046417012	0.371184675

Figure S2. Inter-day precision of AA+DPPH reaction mixture at different concentrations analyzed by applying the established LC-DPPH method. Rt represents the retention time (min). PA describes the peak areas detected from the chromatograms.

AA + DPPH (4 ppm)	No	Rt	PA
Run	1	24.89	3574401.71
	2	24.87	3555565.76
	3	24.88	3593922.58
Mean		24.875	3574744.17
SD		0.011726039	19179.93873
%RSD		0.047139857	0.536540178

AA + DPPH (8 ppm)	No	Rt	PA
Run	1	24.87	3044619.13
	2	24.87	3086126.01
	3	24.85	3092749.83
Mean		24.86	3089437.92
SD		0.012247449	31864.25452
%RSD		0.049265683	1.031393261

AA + DPPH (16 ppm)	No	Rt	PA
Run	1	24.86	2453861.35
	2	24.86	2508930.26
	3	24.87	2448855.84
Mean		24.865	2478893.05
SD		0.006123724	34864.4086
%RSD		0.024627888	1.406450698

Figure 3. Intra-day precision of AA+DPPH reaction mixture at different concentrations analyzed by applying the established LC-DPPH method. Rt represents the retention time (min). PA describes the peak areas detected from the chromatograms.

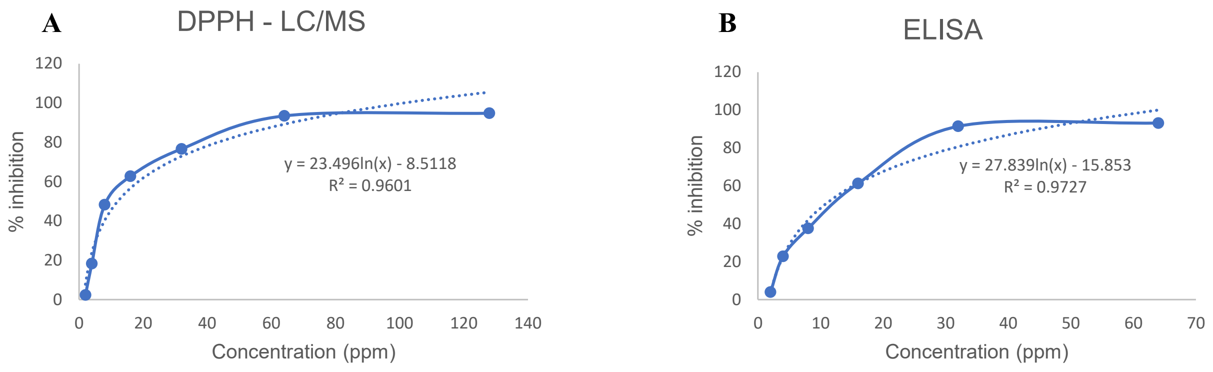

Figure S4. Regress equation of ascorbic acid determined by LC-DPPH (A) and Elisa assay (B).

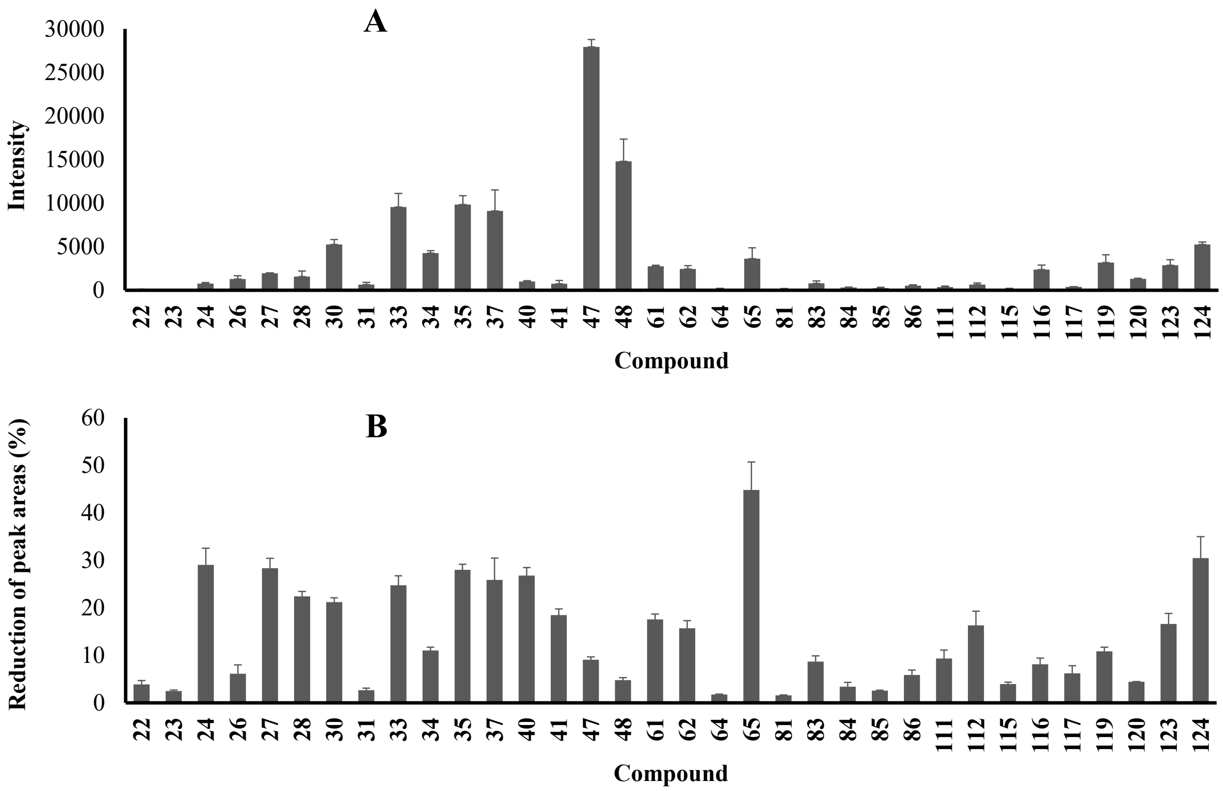

Figure S5. Composition (A) and reduction (%, B) of peak areas of active DPPH components from EtOH extract.

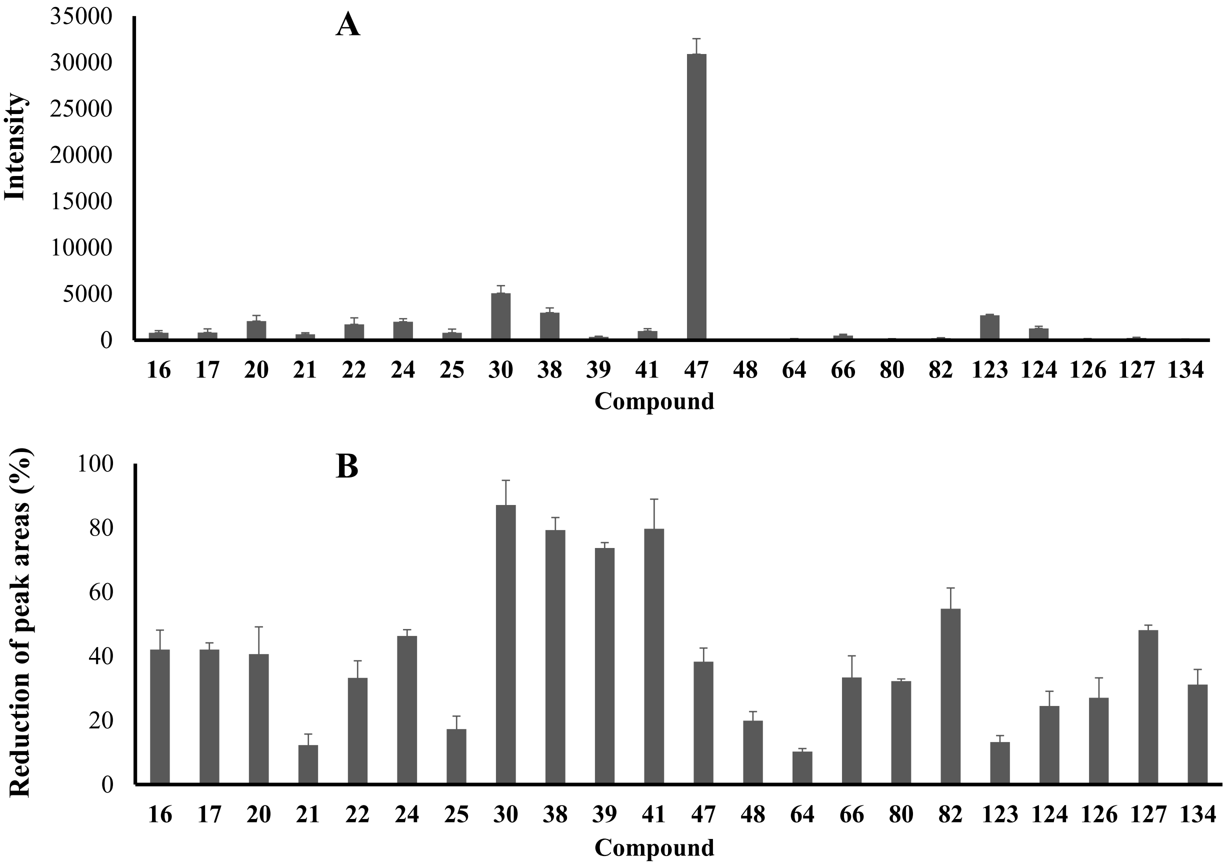

Figure S6. Composition (A) and reduction (%, B) of peak areas of active DPPH components from water extract.

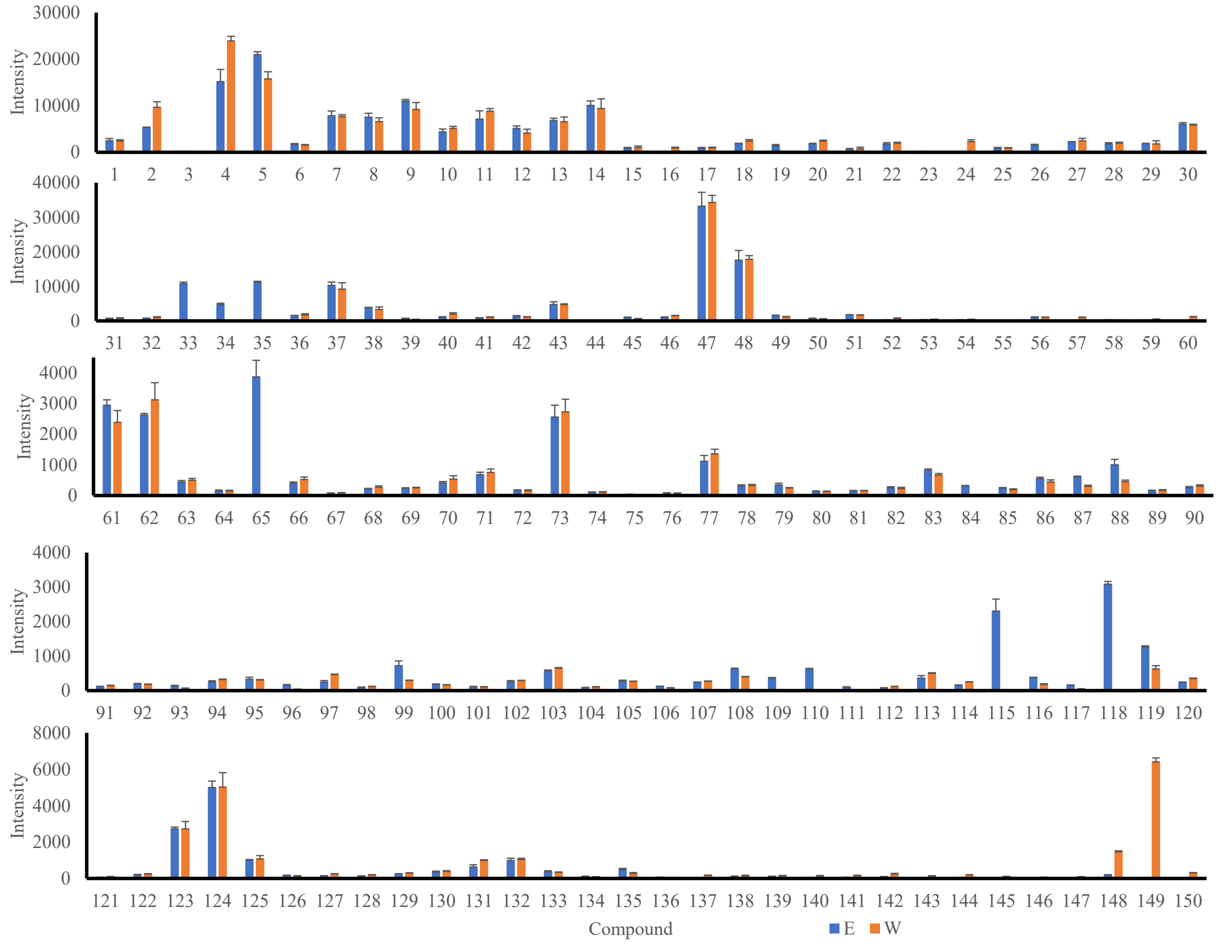

Figure S7. Peak areas of compounds identified from *P. ussuriensis* extracts.

Table S1. Identification of components from SDB fractions.

No.	Compound	RT (min)	Formula	Adduct	*m/z* (Da)	Error (mDa)	Confidence Levels	Class
1	Unknown	0.665	-	$[M+H]^+$	241.9996	-	-	-
2	Unknown	0.708	-	$[M+H]^+$	219.0265	-	-	-
3	Unknown	0.739	-	$[M+H]^+$	320.8663	-	-	-
4	Mannitol	0.812	$C_9H_{10}O_5$	[M+K]+	221.0421	0.1220	1.006	Alcohols
5	L-Proline	0.877	$C_5H_9NO_2$	$[M+H]^+$	116.0706	0.0230	1.774	Alkaloids
6	Sucrose	0.887	$C_{12}H_{22}O_{11}$	$[M+H-H_2O]^+$	325.1130	0.7020	1.344	Disaccharides
7	(*Z*)-2-methyl-4-[(2*R*,3*R*,4*S*,5*S*,6*R*)-3,4,5-trihydroxy-6-(hydroxymethyl)oxan-2-yl]oxybut-2-enenitrile	0.940	$C_{11}H_{17}NO_6$	$[M+H]^+$	260.1128	0.0000	0.891	Alkaloids
8	2-(5,6-dihydroxy-3-methoxycarbonylcyclohex-3-en-1-yl)oxypropanoic acid	0.940	$C_{11}H_{16}O_7$	$[M+NH_4]^+$	278.1234	0.0000	0.500	Organic acids
9	Mycosporine serinol	1.063	$C_{11}H_{19}NO_6$	$[M+H]^+$	262.1285	0.5190	1.272	Alkaloids
10	Unknown	1.235	$C_{12}H_{20}N_2O_3$	$[M+H]^+$	241.1547	0.0059	-	-
11	Adenosine	1.235	$C_{10}H_{13}N_5O_4$	$[M+H]^+$	268.1040	0.0000	1.680	Alkaloids
12	Unknown	1.303	-	$[M+H]^+$	276.1442	-	-	-
13	Unknown	1.303	-	$[M+H]^+$	294.1548	-	-	-
14	Norleucine	1.313	$C_6H_{13}NO_2$	$[M+H]^+$	132.1019	0.0150	0.967	Fatty acids
15	Kinetin riboside	1.559	$C_{15}H_{17}N_5O_5$	$[M+H]^+$	348.1290	1.2820	0.484	Alkaloids
16	(2a*S*,5a*R*,7*R*,8a*R*)-6-Hydroxy-6-[2-(2-hydroxy-5-oxo-2,5-dihydro-3-furanyl)ethyl]-2a,5a,7-trimethyldecahydro-2H-naphtho[1,8-bc]furan-2-one*	2.093	$C_{20}H_{28}O_6$	$[M+H]^+$	365.1919	3.9670	0.609	Terpenoids
17	Picraquassioside D*	2.120	$C_{13}H_{18}O_8$	$[M+Na]^+$	325.0895	0.0920	1.177	Glycosides
18	Benzyl glucopyranoside	2.337	$C_{12}H_{20}O_8$	$[M+H+CH_3CHO]^+$	315.1051	2.8990	1.666	Glycosides

Table S1. Cont.

No.	Compound	RT (min)	Fomula	Adduct	*m/z* (Da)	Error (mDa)	Confidence Levels	Class
19	Benzanthrone	2.347	$C_{17}H_{10}O$	[M+H]+	231.0839	3.5100	1.539	Anthracenes
20	(3R)-4,4-Dimethyl-2-oxotetrahydro-3-furanyl β-D-glucopyranoside*	2.468	$C_{12}H_{20}O_8$	[M+Na]+	315.1051	0.0610	1.666	Glycosides
21	Picrotin*	2.524	$C_{15}H_{18}O_7$	[M+H]+	311.1126	0.0920	1.007	Terpenoids
22	Riboflavin*	2.674	$C_{17}H_{20}N_4O_6$	[M+Na]+	399.1262	0.0920	1.541	Alkaloids
23	Saccatoside	3.613	$C_{30}H_{38}O_{16}$	[M+H]+	655.2211	2.1360	0.474	Terpenoids
24	Unknown*	3.630	-	[M+H]+	392.0953	-	-	-
25	2-Hydroxy-4-(2-hydroxyethyl)phenyl *β*-D-glucopyranoside*	3.655	$C_{14}H_{20}O_8$	[M+Na]+	339.1051	0.0920	1.482	Phenols
26	Dianthoside*	3.692	$C_{12}H_{16}O_8$	[M+H]+	289.0894	2.3800	0.446	Glycosides
27	Pantothenic acid*	4.019	$C_9H_{17}NO_5$	[M+H]+	220.1179	0.0150	1.381	Alkaloids
28	Methocarbamol*	4.019	$C_{11}H_{15}NO_5$	[M+H]+	242.0999	2.4110	0.955	Alkaloids
29	5,7-dihydroxy-2-methyl-8-[(2*S*,3*R*,4*R*,5*S*,6*R*)-3,4,5-trihydroxy-6-(hydroxymethyl)oxan-2-yl]chromen-4-one	4.292	$C_{16}H_{18}O_9$	[M+H]+	355.1000	2.3500	1.014	Chromones
30	Ellagic acid*	4.465	$C_{14}H_6O_8$	[M+H]+	303.1051	0.8850	0.448	Tannins
31	4-(Hexopyranosyloxy)-3-methoxybenzoic acid*	4.544	$C_{14}H_{18}O_9$	[M+Na]+	353.0844	0.0920	0.522	Polyphenols
32	(2*R*,3*S*,4*S*,5*R*,6*R*)-2-[[(2*R*,3*R*,4*R*)-3,4-dihydroxy-4-(hydroxymethyl)oxolan-2-yl]oxymethyl]-6-[2-(4-hydroxyphenyl)ethoxy]oxane-3,4,5-triol	4.725	$C_{19}H_{28}O_{11}$	[M+H]+	433.1680	2.4410	0.446	Glycosides
33	Iretol*	5.641	$C_7H_8O_4$	[M+H]+	157.0495	0.0000	0.500	Phenols
34	Ribitol*	5.641	$C_5H_{12}O_5$	[M+Na]+	175.0601	0.1070	1.016	Glycosides
35	Unknown*	5.669	-	[M+H]+	243.0474	-	-	-

Table S1. Cont.

No.	Compound	RT (min)	Formula	Adduct	*m/z* (Da)	Error (mDa)	Confidence Levels	Class
36	Geniposidic Acid	5.778	$C_{16}H_{22}O_{10}$	$[M+Na]^+$	397.1105	0.0310	1.593	Glycosides
37	Unknown*	5.896	-	$[M+H]^+$	188.0706	-	-	-
38	Tryptophan*	5.917	$C_{11}H_{12}N_2O_2$	$[M+H]^+$	205.0971	0.0460	1.472	Alkaloids
39	2-(1,2-dihydroxyethyl)-3-[(*E*)-3-(3,4-dihydroxyphenyl)prop-2-enoyl]oxy-4-hydroxy-3,4-dihydro-2H-pyran-6-carboxylic acid*	6.429	$C_{17}H_{18}O_{10}$	$[M+H]^+$	383.0949	2.3190	0.449	Organic acids
40	Secologanol*	6.696	$C_{17}H_{26}O_{10}$	$[M+Na]^+$	413.1418	0.0000	1.358	Glycosides
41	8-Hydroxy-6,7-dimethoxychromen-2-one*	7.212	$C_{11}H_{10}O_5$	$[M+H]^+$	223.0577	2.3960	1.143	Coumarins
42	Methylxanthoxylin	7.884	$C_{11}H_{14}O_4$	$[M+H]^+$	211.0965	0.5040	1.016	Phenols
43	Hydrastine	8.545	$C_{21}H_{21}NO_6$	$[M+H]^+$	406.1344	4.6690	0.323	Alkaloids
44	3,4-di-*O*-caffeoylquinic acid	9.348	$C_{25}H_{24}O_{12}$	$[M+H]^+$	517.1551	0.0211	0.890	Quinic acid
45	Unknown	9.646	-	$[M+H]^+$	319.1514	-	-	-
46	Unknown	9.677	-	$[M+H]^+$	660.1710	-	-	-
47	7-hydroxy-coumarin*	10.170	$C_9H_6O_3$	$[M+H]^+$	163.03894	0.0310	1.580	Coumarins
48	Chlorogenic acid*	10.187	$C_{16}H_{18}O_9$	$[M+H]^+$	355.1024	0.0014	0.988	Polyphenol
49	Grandidentoside	10.802	$C_{21}H_{28}O_{10}$	$[M+Na]^+$	463.1575	0.0000	1.627	Glycosides
50	Periplanetin	11.000	$C_{13}H_{16}O_7$	$[M+Na]^+$	307.0789	0.0610	1.133	Glycosides
51	Junipediol B 8-O-glucoside	11.086	$C_{16}H_{22}O_9$	$[M+Na]^+$	381.1157	0.0920	1.646	Glycosides
52	Chicoric acid	11.203	$C_{22}H_{18}O_{12}$	$[M+H-C_{13}H_{12}O_9]^+$	163.0390	0.0000	0.900	Organic acids
53	7-hydroxy-5-methyl-2-(2-oxopropyl)-8-[3,4,5-trihydroxy-6-(hydroxymethyl)oxan-2-yl]chromen-4-one	11.395	$C_{19}H_{22}O_9$	$[M+H]^+$	395.1313	2.3800	1.035	Glycosides

Table S1. Cont.

No.	Compound	RT (min)	Formula	Adduct	*m/z* (Da)	Error (mDa)	Confidence Levels	Class
54	Syringin	11.400	$C_{17}H_{24}O_9$	$[M+NH_4]^+$	390.1759	0.0610	1.009	Terpenoids
55	Methyl 6-hydroxy-7-methyl-1-[(2S,3S,4R,5R,6S)-3,4,5-trihydroxy-6-(hydroxymethyl)oxan-2-yl]oxy-1,4a,5,6,7,7a-hexahydrocyclopenta[c]pyran-4-carboxylate	11.612	$C_{24}H_{30}O_{13}$	$[M+NH_4]^+$	544.2026	0.1220	1.414	Terpenoids
56	(2*S*,3*R*,4*S*,5*S*,6*R*)-2-[[(1*S*,4a*R*,7a*S*)-7-(hydroxymethyl)-1,4a,5,7a-tetrahydrocyclopenta[c]pyran-1-yl]oxy]-6-(hydroxymethyl)oxane-3,4,5-triol	12.368	$C_{15}H_{22}O_8$	$[M+H]^+$	331.1364	2.3190	1.472	Terpenoids
57	Procyanidin B2	12.469	$C_{30}H_{26}O_{12}$	$[M+H]^+$	579.1498	0.1220	0.449	Polyphenol
58	Cichorioside B	12.564	$C_{21}H_{28}O_{10}$	$[M+H]^+$	441.1731	2.4410	1.358	Glycosides
59	(+)-Genipin	13.480	$C_{11}H_{14}O_5$	$[M+H]^+$	227.0914	0.0150	1.143	Terpenoids
60	(-)-Epicatechin	13.720	$C_{15}H_{14}O_6$	$[M+H]^+$	291.0863	0.0003	1.016	Polyphenol
61	4-[4-(*β*-D-glucopyranosyloxy)-2-hydroxy-2,6,6-trimethylcyclohexylidene]-3-buten-2-one*	13.934	$C_{19}H_{30}O_8$	$[M+Na]^+$	409.1833	0.0610	0.323	Terpenoids
62	Unknown*	13.937	-	$[M+H]^+$	207.1379	-	-	Unknown
63	Roseoside	13.937	$C_{19}H_{30}O_8$	$[M+H]^+$	387.2014	0.0610	-	Glycosides
64	Cinnamoside*	14.433	$C_{24}H_{38}O_{12}$	$[M+Na]^+$	541.2256	0.0610	-	Glycosides
65	Unknown	14.476	-	$[M+H]^+$	247.0812	-	1.580	-
66	9,14-Dimethyl-5-methylidene-3,13-dioxatetracyclo[8.4.0.02,6.012,14]tetradec-9-ene-4,11-dione*	14.481	$C_{15}H_{16}O_4$	$[M+H]^+$	261.1121	0.0000	0.988	Terpenoids
67	(+)-Lyoniresinol 9-glucoside	14.815	$C_{28}H_{38}O_{13}$	$[M+NH_4]^+$	600.2651	0.0610	1.627	Glycosides
68	Unknown	14.881	-	$[M+H]^+$	388.2540	-	1.133	-
69	Icariside B8	14.952	$C_{19}H_{32}O_8$	$[M+Na]^+$	411.1990	0.0310	1.646	Terpenoids
70	(*E*)-3-[4-methoxy-2-[(2*S*,3*R*,4*S*,5*S*,6*R*)-3,4,5-trihydroxy-6-(hydroxymethyl)oxan-2-yl]oxyphenyl]prop-2-enoic acid	15.903	$C_{16}H_{20}O_9$	$[M+H]^+$	357.1157	2.3190	0.900	Phenylpropanoids
71	Unknown	15.908	-	$[M+H]^+$	352.1603	-	1.035	-

Table S1. Cont.

No.	Compound	RT (min)	Formula	Adduct	*m/z* (Da)	Error (mDa)	Confidence Levels	Class
72	3,4-Dihydroxyallylbenzene 3,4-di-*O*-glucoside	15.967	$C_{21}H_{30}O_{12}$	$[M+NH_4]^+$	492.2076	0.0000	1.009	Phenylpropanoids
73	Unknown	16.109	-	$[M+H]^+$	343.1364	-	1.414	-
74	Acarbose	16.109	$C_{25}H_{43}NO_{18}$	$[M+NH_4]^+$	663.2842	2.4410	1.472	Glycosides
75	5,7-dihydroxy-2-[3-hydroxy-4-[(2*S*,3*R*,4*S*,5*S*,6*R*)-3,4,5-trihydroxy-6-(hydroxymethyl)oxan-2-yl]oxyphenyl]-3-[(2*S*,3*R*,4*S*,5*S*,6*R*)-3,4,5-trihydroxy-6-(hydroxymethyl)oxan-2-yl]oxychromen-4-one	16.416	$C_{27}H_{30}O_{17}$	$[M+H]^+$	627.1557	0.0007	0.959	Flavonoids
76	3-[(2*S*,3*R*,4*S*,5*S*,6*R*)-6-[[(2*R*,3*R*,4*R*,5*R*,6*S*)-3-[(2*S*,3*R*,4*R*)-3,4-dihydroxy-4-(hydroxymethyl)oxolan-2-yl]oxy-4,5-dihydroxy-6-methyloxan-2-yl]oxymethyl]-3,4,5-trihydroxyoxan-2-yl]oxy-2-(3,4-dihydroxyphenyl)-5,7-dihydroxychromen-4-one	16.762	$C_{32}H_{38}O_{20}$	$[M+H]^+$	743.2035	0.0034	0.917	Flavonoids
77	Unknown	16.829	-	$[M+H]^+$	205.1434	-	-	-
78	Gallomyrtucommulone C	16.998	$C_{27}H_{36}O_{13}$	$[M+NH_4]^+$	586.2496	0.2440	0.500	Flavonoids
79	2-(3,4-dihydroxyphenyl)-5,7-dihydroxy-3-[(2*S*,3*R*,4*S*,5*S*,6*R*)-3,4,5-trihydroxy-6-[[(2*S*,3*R*,4*S*,5*R*)-3,4,5-trihydroxyoxan-2-yl]oxymethyl]oxan-2-yl]oxychromen-4-one	17.120	$C_{26}H_{28}O_{16}$	$[M+H]^+$	597.1452	0.0002	0.931	Flavonoids
80	Unknown	17.276	$C_{19}H_{27}NO_{11}$	$[M+H]^+$	446.1656	0.0377	-	-
81	Rhodioloside*	17.296	$C_{14}H_{20}O_7$	$[M+H]^+$	301.1258	2.3500	1.276	Glycosides
82	Unknown	17.316	$C_{26}H_{39}NO_{11}$	$[M+H]^+$	542.2596	0.0560	-	-
83	Junipediol A*	17.323	$C_{16}H_{24}O_9$	$[M+Na]^+$	383.1314	0.0920		Glycosides
84	5-Hydroxy-2-[2-hydroxy-3-[(2S,3R,4S,5S,6R)-3,4,5-trihydroxy-6-(hydroxymethyl)oxan-2-yl]oxyphenyl]-7,8-dimethoxychromen-4-one*	17.323	$C_{23}H_{24}O_{12}$	$[M+Na]^+$	515.1185	2.5020		Flavonoids
85	Guaiphenesin*	17.333	$C_{10}H_{14}O_4$	$[M+H]^+$	199.0965	0.0460	1.030	Phenols
86	7-methyl-1-[(2*S*,3*R*,4*S*,5*S*,6*R*)-3,4,5-trihydroxy-6-(hydroxymethyl)oxan-2-yl]oxy-1,4a,5,6,7,7a-hexahydrocyclopenta[c]pyran-4-carboxylic acid*	17.333	$C_{16}H_{24}O_9$	$[M+NH4]^+$	378.1759	0.0610	1.255	Terpenoids
87	Fumitremorgin A	17.350	$C_{32}H_{41}N_3O_7$	$[M+Na]^+$	602.2811	2.6250	0.455	Alkaloids

Table S1. Cont.

No.	Compound	RT (min)	Formula	Adduct	m/z (Da)	Error (mDa)	Confidence Levels	Class
88	Ouabain	17.350	$C_{29}H_{44}O_{12}$	$[M+Na]^+$	607.2364	1.9530	0.475	Glycosides
89	8-Hydroxypinoresinol-4'-O-*β*-D-Glucopyranoside	17.489	$C_{26}H_{32}O_{12}$	$[M+NH_4]^+$	554.2233	0.0000	1.558	Glycosides
90	1,2,3-trimethoxy-5-[2-[4-(3-methylbut-2-enoxy)phenyl]ethyl]benzene	17.529	$C_{22}H_{28}O_4$	$[M+Na]^+$	379.1865	1.4950	0.478	Phenolics
91	Unknown	17.535	-	$[M+H]^+$	520.3329	-	-	-
92	Rutin	17.593	$C_{27}H_{30}O_{16}$	$[M+H]^+$	611.1616	0.9770	1.661	Flavonoids
93	7-hydroxy-2-(4-hydroxyphenyl)-8-[(2*S*,3*R*,4*R*,5*S*,6*R*)-3,4,5-trihydroxy-6-(hydroxymethyl)oxan-2-yl]chromen-4-one	17.619	$C_{21}H_{20}O_9$	$[M+H]^+$	417.1157	2.3190	1.050	Flavonoids
94	Clerodin	17.634	$C_{24}H_{34}O_7$	$[M+Na]^+$	457.2408	1.2510	0.485	Terpenoids
95	Quercetin	17.659	$C_{15}H_{10}O_7$	$[M+H]^+$	303.0499	0.0310	1.476	Flavonoids
96	Unknown	17.673	-	$[M+H]^+$	554.2708	-	-	-
97	Unknown	17.708	-	$[M+H]^+$	329.1596	-	-	-
98	Hyperoside	17.767	$C_{21}H_{20}O_{12}$	$[M+H]^+$	465.1028	0.0000	1.792	Flavonoids
99	4-[3-[(2*S*,3*R*,4*S*,5*S*,6*R*)-6-[[(2*R*,3*R*,4*R*)-3,4-dihydroxy-4-(hydroxymethyl)oxolan-2-yl]oxymethyl]-3,4,5-trihydroxyoxan-2-yl]oxy-2-hydroxy-3-methylbutoxy]furo[3,2-g]chromen-7-one	17.787	$C_{27}H_{34}O_{15}$	$[M+H]^+$	599.1973	0.2440	0.500	Glycosides
100	Asperulosidic Acid	17.792	$C_{18}H_{24}O_{12}$	$[M+H-H_2O]^+$	415.1235	0.0000	0.500	Terpenoids
101	Protopine	17.827	$C_{20}H_{19}NO_5$	$[M+H]^+$	354.1760	1.6780	0.473	Alkaloids
102	Unknown	17.906	$C_{19}H_{30}N_6O_7$	$[M+H]^+$	455.2252	0.3765	-	-
103	2-Phenylethyl 3-*O*-(4-carboxy-3-hydroxy-3-methylbutanoyl)-*β*-D-glucopyranoside	17.946	$C_{20}H_{28}O_{10}$	$[M+H]^+$	429.1732	2.3800	0.996	Glycosides
104	(*E*)-Coniferin	18.018	$C_{16}H_{22}O_8$	$[M+Na]^+$	365.1208	0.0610	1.447	Glycosides
105	3-[[(2*R*,3*S*,4*S*,5*R*,6*S*)-6-[2-(3,4-dihydroxyphenyl)-5,7-dihydroxy-4-oxochromen-3-yl]oxy-3,4,5-trihydroxyoxan-2-yl]methoxy]-3-oxopropanoic acid	18.058	$C_{24}H_{22}O_{15}$	$[M+H]^+$	551.1032	0.0610	1.529	Flavonoids

Table S1. Cont.

No.	Compound	RT (min)	Formula	Adduct	*m/z* (Da)	Error (mDa)	Confidence Levels	Class
106	[(1*R*,2*E*,8*S*,10*R*,11*S*)-10,11-dihydroxy-6-(methoxymethyl)-1,10-dimethyl-5-oxo-4,14-dioxatricyclo[9.2.1.03,7]tetradeca-2,6-dien-8-yl] 2-methylprop-2-enoate	18.102	$C_{20}H_{26}O_8$	$[M+H]^+$	395.1677	1.9840	0.972	Terpenoids
107	Isorhamnetin-3-galactoside-6-rhamnoside	18.156	$C_{28}H_{32}O_{16}$	$[M+H]^+$	625.1765	0.1830	1.582	Flavonoids
108	Unknown	18.195	-	$[M+H]^+$	343.2092	-	0.449	-
109	9-methoxy-7-[4-[(2*S*,3*R*,4*S*,5*S*,6*R*)-3,4,5-trihydroxy-6-(hydroxymethyl)oxan-2-yl]oxyphenyl]-[1,3]dioxolo[4,5-g]chromen-8-one*	18.195	$C_{23}H_{22}O_{11}$	$[M+Na]^+$	497.1079	2.5020	0.780	Flavonoids
110	5-Hydroxy-7-[3,4,5-trihydroxy-6-(hydroxymethyl)oxan-2-yl]oxy-2-[4-(3,4,5-trihydroxy-6-methyloxan-2-yl)oxyphenyl]chromen-4-one	18.221	$C_{27}H_{30}O_{14}$	$[M+H]^+$	579.1709	0.0001	0.976	Flavonoids
111	(3R,5R)-3,5-Bis[[(E)-3-(3,4-dihydroxyphenyl)prop-2-enoyl]oxy]-1,4-dihydroxycyclohexane-1-carboxylic acid	18.245	$C_{25}H_{24}O_{12}$	$[M+H]^+$	517.1340	0.0018	0.974	Quinic acid
112	3,5-Dicaffeoylquinic acid	18.250	$C_{25}H_{24}O_{12}$	$[M+H-H_2O]^+$	499.1236	0.1220	1.385	Quinic acid
113	Methyl (2*S*,4a*S*,6a*R*,7*R*,10a*R*,10b*R*)-2-(3-furanyl)-1,4,4a,5,6,6a,7,10,10a,10b-decahydro-7-hydroxy-6a,10b-dimethyl-4-oxo-2H-naphtho[2,1-c]pyran-7-carboxylate*	18.225	$C_{21}H_{26}O_6$	$[M+Na]^+$	397.1646	2.4720	1.596	Terpenoids
114	5-hydroxy-2-(4-hydroxyphenyl)-7-[(2S,3R,4S,5S,6R)-3,4,5-trihydroxy-6-[[(2R,3R,4R,5R,6S)-3,4,5-trihydroxy-6-methyloxan-2-yl]oxymethyl]oxan-2-yl]oxy-2,3-dihydrochromen-4-one*	18.275	$C_{27}H_{32}O_{14}$	$[M+H]^+$	581.1866	0.1830	1.377	Flavonoids
115	Isorhamnetin 3-*O*-glucoside*	18.285	$C_{22}H_{22}O_{12}$	$[M+H]^+$	479.1184	0.0024	0.950	Flavonoids
116	(2S,3R,4S,5S,6R)-2-[4-[(3S,3aR,6S,6aR)-3-(4-hydroxy-3,5-dimethoxyphenyl)-1,3,3a,4,6,6a-hexahydrofuro[3,4-c]furan-6-yl]-2,6-dimethoxyphenoxy]-6-(hydroxymethyl)oxane-3,4,5-triol*	18.314	$C_{28}H_{36}O_{13}$	$[M+NH_4]^+$	598.2496	0.1830		Glycosides
117	(+)-syringaresinol β-D-glucoside	18.314	$C_{28}H_{36}O_{13}$	$[M+NH_4]^+$	598.2496	0.1830	1.762	Glycosides
118	Naringenin*	18.320	$C_{15}H_{12}O_5$	$[M+H]^+$	273.0757	0.0920	0.974	Flavonoids
119	Isorhamnetin*	18.364	$C_{16}H_{12}O_7$	$[M+H]^+$	317.0657	0.0043	0.953	Flavonoids
120	Unknown	18.416	-	$[M+H]^+$	476.2128	-	0.777	-
121	(2*E*,4*E*,8*E*)-7,13-dihydroxy-4,8,12-trimethyltetradeca-2,4,8-trienoic acid	18.440	$C_{17}H_{28}O_4$	$[M+H]^+$	297.2038	2.2580	0.965	Fatty acids

Table S1. Cont.

No.	Compound	RT (min)	Formula	Adduct	*m/z* (Da)	Error (mDa)	Confidence Levels	Class
122	Cyclopentaneacetic acid	18.555	$C_{18}H_{30}O_8$	$[M+Na]^+$	397.1834	0.0920	1.631	Glycosides
123	(5*R*)-5-hydroxy-1-(4-hydroxy-3-methoxyphenyl)decan-3-one*	18.691	$C_{17}H_{26}O_4$	$[M+H]^+$	295.1881	2.3500	0.448	Phenols
124	(4*S*,4a*R*)-4-(Hydroxymethyl)-3,4a,8,8-tetramethyl-4a,5,6,7,8,8a-hexahydro-1(4H)-naphthalenone*	18.695	$C_{15}H_{24}O_2$	$[M+H]^+$	237.1849	0.0150	1.141	Terpenoids
125	9-hydroxyageraphorone	18.696	$C_{15}H_{24}O_2$	$[M+H-H_2O]^+$	219.1744	0.0000	1.054	Terpenoids
126	Acantrifoside E*	18.856	$C_{17}H_{24}O_8$	$[M+Na]^+$	379.1364	0.0610	1.620	Glycosides
127	6-Hydroxy-2,6,10,10-tetramethyl-1-oxaspiro[4.5]dec-8-yl 6-*O*-[(2*R*,3*R*,4*R*)-3,4-dihydroxy-4-(hydroxymethyl)tetrahydro-2-furanyl]-*β*-D-glucopyranoside*	18.856	$C_{24}H_{42}O_{12}$	$[M+H-H_2O]^+$	505.2658	1.4950	1.008	Terpenoids
128	Unknown	18.912	-	$[M+H]^+$	764.3127	-	-	-
129	5,5,8a-Trimethyl-1,4,4a,5,6,7,8,8a-octahydronaphthalene-1,2-dicarbaldehyde	18.937	$C_{15}H_{22}O_2$	$[M+H+CH_3COOH]^+$	293.1723	0.6710	1.652	Terpenoids
130	(1*β*,2*α*,9*ξ*,11*β*,12*α*,15*β*)-1,2,11,12,14,15-hexahydroxy-Picras-4-en-16-one	19.017	$C_{20}H_{30}O_8$	$[M+H]^+$	399.1990	2.3500	0.949	Terpenoids
131	Unknown	19.107	$C_{19}H_{30}N_6O_6$	$[M+H]^+$	439.2303	0.3265	-	-
132	2-{[6-*O*-(6-Deoxy-*α*-L-mannopyranosyl)-*β*-D-glucopyranosyl]oxy}-3-methylbutanenitrile	19.385	$C_{17}H_{29}NO_{10}$	$[M+Na]^+$	430.1708	2.4410	0.444	Alkaloids
133	4-[(*E*)-3-[(2*R*,3*R*,4*S*,5*S*,6*R*)-3-[(2*S*,3*R*,4*R*)-3,4-dihydroxy-4-(hydroxymethyl)oxolan-2-yl]oxy-4,5-dihydroxy-6-(hydroxymethyl)oxan-2-yl]oxybut-1-enyl]-3,5,5-trimethylcyclohex-2-en-1-one	19.478	$C_{24}H_{38}O_{11}$	$[M+NH_4]^+$	520.2753	0.0610	0.500	Glycosides
134	7-hydroxy-3-[4-hydroxy-3-(3-methylbut-2-enyl)phenyl]chromen-4-one*	19.942	$C_{20}H_{18}O_4$	$[2M+Na]^+$	667.2367	6.7140	0.304	Flavonoids
135	Unknown	20.137	-	$[M+H]^+$	607.2153	-	-	-
136	1-*O*-[(2*α*,3*β*,5*ξ*,9*ξ*,18*ξ*)-2,3,19-trihydroxy-28-oxours-12-en-28-yl]- *β*-D-Glucopyranose	20.255	$C_{36}H_{58}O_{10}$	$[M+NH_4]^+$	668.4373	0.0003	0.860	Glycosides
137	Berberine	20.441	$C_{20}H_{18}NO_4$	$[M+H]^+$	337.1984	3.9060	0.370	Alkaloids
138	Unknown	20.572	$C_{13}H_{23}NO_2$	$[M+H]^+$	226.1801	0.1017	-	-

Table S1. Cont.

No.	Compound	RT (min)	Formula	Adduct	*m/z* (Da)	Error (mDa)	Confidence Levels	Class
139	Parthenolide	20.572	$C_{15}H_{20}O_3$	[M+NH4]+	266.1726	2.3800	1.105	Isoprenoids
140	(10*E*,12*E*)-9-hydroxyoctadeca-10,12-dienoic acid	20.602	$C_{18}H_{32}O_3$	$[M+H-H_2O]^+$	279.2319	0.0610	1.065	Fatty Acids
141	8-hydroxy-8-(3-octyloxiran-2-yl)octanoic acid	20.602	$C_{18}H_{34}O_4$	$[M+H-H_2O]^+$	297.2425	0.0610	1.071	Fatty Acids
142	Unknown	20.602	-	$[M+H]^+$	355.2456	-	-	-
143	Unknown	20.644	-	$[M+H]^+$	555.2203	-	-	-
144	Phytosphingosine	21.989	$C_{18}H_{39}NO_3$	$[M+H]^+$	318.3003	0.0310	1.218	Alkaloids
145	Unknown	22.186	$C_{19}H_{41}NO_2$	$[M+H]^+$	316.3211	0.1000	-	-
146	Tormentic Acid	22.661	$C_{30}H_{48}O_5$	$[M+H]^+$	489.3574	0.0004	0.788	Terpenoids
147	(2α,3β,5ξ,9ξ,19α)-2,3,19-trihydroxy-Olean-12-en-28-oic acid	22.672	$C_{30}H_{48}O_5$	$[M+H-H_2O]^+$	471.3469	0.0009	0.761	Terpenoids
148	Unknown	23.887	-	$[M+H]^+$	452.3218	-	-	-
149	5,7-dihydroxy-6-[3,4,5-trihydroxy-6-(hydroxymethyl)oxan-2-yl]-8-[3,4,5-trihydroxy-6-(hydroxymethyl)oxan-2-yl]oxychromen-2-one	25.445	$C_{21}H_{26}O_{15}$	$[M+NH_4]^+$	536.1656	4.5780	0.347	Glycosides
150	Unknown	27.406	-	$[M+H]^+$	612.3722	-	-	-
*: Compound has an antioxidant activity, evaluated by LC-DPPH assay.								

Table S2. Spectra of identified compound has an antioxidant activity, evaluated by LC-DPPH.

No.	Compound	RT (min)	Formula	M/Z	Error (mDa)	Note	Peak reduction Mean ± SD
21	Picrotin*	2.524	$C_{15}H_{18}O_7$	311.1126	0.0920	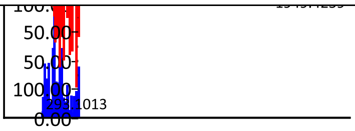	12.31 ± 0.33 (W)
30	Ellagic acid*	4.465	$C_{14}H_6O_8$	303.1051	0.8850	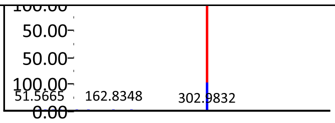	87.10 ± 6.62 (W)
47	7-hydroxy-coumarin*	10.170	$C_9H_6O_3$	163.0389	0.0310	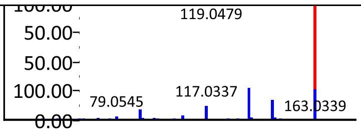	38.27 ± 0.72 (W)
48	Chlorogenic acid*	10.187	$C_{16}H_{18}O_9$	355.1024	0.0014	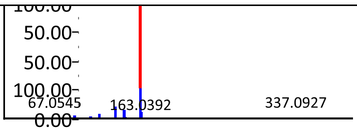	19.93 ± 2.33 (W)
61	4-[4-(*β*-D-glucopyranosyloxy)-2-hydroxy-2,6,6-trimethylcyclohexylidene]-3-buten-2-one*	13.934	$C_{19}H_{30}O_8$	409.1833	0.0610	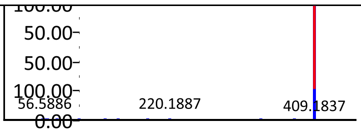	17.59 ± 0.67 (E)

66	9,14-Dimethyl-5-methylidene-3,13-dioxatetracyclo[8.4.0.02,6.012,14]tetradec-9-ene-4,11-dione*	14.481	$C_{15}H_{16}O_4$	261.1121	0.0000	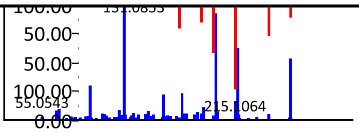	33.42 ± 1.83 (W)
83	Junipediol A*	17.323	$C_{16}H_{24}O_9$	383.1314	0.0920	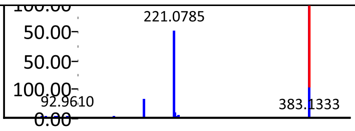	54.78 ± 1.76 (W)
115	Isorhamnetin 3-***O***-glucoside*	18.285	$C_{22}H_{22}O_{12}$	479.1184	0.0024	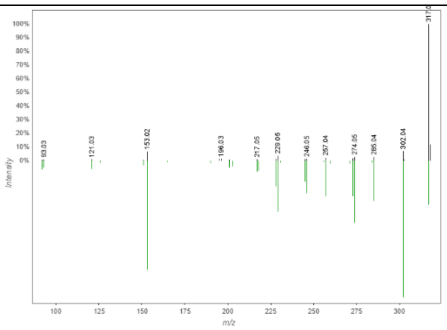	6.21 ± 0.24 (E)
123	(5***R***)-5-hydroxy-1-(4-hydroxy-3-methoxyphenyl)decan-3-one*	18.691	$C_{17}H_{26}O_4$	295.1881	2.3500	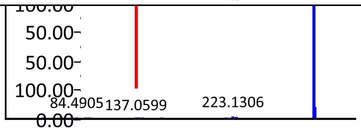	13.23 ± 0.64 (W)
124	(4***S***,4a***R***)-4-(Hydroxymethyl)-3,4a,8,8-tetramethyl-4a,5,6,7,8,8a-hexahydro-1(4H)-naphthalenone*	18.695	$C_{15}H_{24}O_2$	237.1849	0.0150	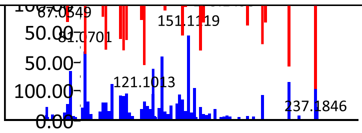	24.47 ± 1.19 (W)

*W: Water extract
E: Ethanol extract
